# Designing overall stoichiometric conversions and intervening metabolic reactions

**DOI:** 10.1038/srep16009

**Published:** 2015-11-04

**Authors:** Anupam Chowdhury, Costas D. Maranas

**Affiliations:** 1 Department of Chemical Engineering, The Pennsylvania State University, University Park, PA 16802.

## Abstract

Existing computational tools for *de novo* metabolic pathway assembly, either based on mixed integer linear programming techniques or graph-search applications, generally only find linear pathways connecting the source to the target metabolite. The overall stoichiometry of conversion along with alternate co-reactant (or co-product) combinations is not part of the pathway design. Therefore, global carbon and energy efficiency is in essence fixed with no opportunities to identify more efficient routes for recycling carbon flux closer to the thermodynamic limit. Here, we introduce a two-stage computational procedure that both identifies the optimum overall stoichiometry (i.e., optStoic) and selects for (non-)native reactions (i.e., minRxn/minFlux) that maximize carbon, energy or price efficiency while satisfying thermodynamic feasibility requirements. Implementation for recent pathway design studies identified non-intuitive designs with improved efficiencies. Specifically, multiple alternatives for non-oxidative glycolysis are generated and non-intuitive ways of co-utilizing carbon dioxide with methanol are revealed for the production of C_2+_ metabolites with higher carbon efficiency.

Microbial metabolism describes the full range of enzymatic conversions of carbon substrates to cellular biomass precursors, energy equivalents and biochemical molecules. Metabolic engineering harnesses this metabolic machinery for converting feedstock substrates to a growing range of products^1^,^2^,^3^. Starting with single gene mutations, the range of interventions over the past decade has expanded considerably to enable genome-wide editing and pathway assembly^4^. The use of system and synthetic biology tools have enabled the tunable regulation of genes^5^,^6^,^7^,^8^, assembly of heterologous pathways^9^,^10^ and temporal control of gene expression^11^,^12^. Recent advances in multiplex engineering (e.g., MAGE^13^), efficient genome-editing (e.g., CRISPR-Cas^14^,^15^) and genome-wide regulation of gene expression through small RNAs^16^ have brought closer the dream of “designer cells” that can catalyze any tailor-made stoichiometry-balanced metabolic conversion with high specificity and control^17^. Concurrent with experimental efforts, computational strain design tools relying on stoichiometry^18^,^19^,^20^ along with kinetic expressions^21^,^22^ is increasingly being used to guide the redesign of microbial metabolism. So far, these redesign approaches have mostly concentrated on retrofitting the metabolic capabilities of the production host by preventing carbon loss and ensuring proper redox supply.

Existing computational procedures for the *de novo* pathway design rely on either optimization techniques or graph-search approaches. Linear Programming (LP) and Mixed Integer Linear Programming (MILP) approaches for pathway design, in general, extract a minimal stoichiometry-balanced sub-network that converts a source metabolite to a target chemical with high yield^23^. While early work was restricted to design pathways for small-to-medium size networks^24^,^25^,^26^, recent procedures have reached up to genome-scale size^27^,^28^,^29^ often using the concept of elementary modes^30^ and comprehensive databases of reactions^31^,^32^,^33^,^34^. However, these procedures do not necessarily conform to a previously identified optimal conversion stoichiometry thereby missing the opportunity to optimally recycle intermediates to reach a maximum yield.

Graph-based pathway design approaches^35^ begin from the target chemical and iteratively retrace back a path to the source metabolite using a retrosynthetic algorithm. Using a depth-first search, variations of the k-shortest path approach have been used to design and classify pathways based on their length^36^, negativity of free energy change for each reaction step^37^, or use of intermediate metabolites with the fewest connections^38^. Graph-search approaches can be supplemented with “reaction-rules” that can suggest *de novo* reaction steps between any two metabolites often absent in existing databases (e.g., BNICE^39^, Pertusi *et al.*^40^, GEM-Path^41^). In most cases, the consumption (or production) of cofactors and possibly co-reactants or co-products required for these pathways to operate is not directly tracked^42^. As a result, these procedures are often coupled with a post-processing step where a Flux Balance Analysis (FBA) is performed to select pathways that are stoichiometry-balanced and maximize the production of target chemicals. As with LP or MILP based approaches, generally mostly linear pathway topologies are traced.

Powerful as these approaches have been, their primary shortcoming lies in the fact that they aim to trace pathways connecting a single substrate (A) to a single product (B). However, metabolic conversions do not generally involve linear paths from substrate to product^43^. Most metabolic engineering tasks recruit many more metabolites and reactions than the ones along the main carbon conversion path. Cofactor usage, co-reactant choices as well as stoichiometric ratios are important decision variables during the pathway design stage. It is the overall stoichiometry that globally captures the overall goal rather than just the origin and destination molecules. The term overall stoichiometry/conversion abstracts the global elemental balance sheet for the chemical changes including metabolites, small molecules, ions and free energy equivalents required to achieve the design objective. Meeting this overall stoichiometry often requires the recruitment of many additional metabolites that help recycle carbon and redox resources in highly complex ways. This motivates the development of a stoichiometry and pathway design tool (Fig. 1) that first optimizes the overall stoichiometry (e.g., *aA* + *cC* → *bB* + *dD*) by exploring exhaustively co-reactant/co-product combinations (Step 1). The coefficients in the overall stoichiometry (i.e., *a, b, c* and *d*) are critical as they ultimately define the carbon and energy efficiency of the conversion while meeting thermodynamic feasibility. Following the selection of the overall reaction (i.e., optStoic, see Fig. 1 and Methods), intervening reactions can be identified from a database (Step 2) that links the chosen reactants and products in the desired stoichiometric ratios (i.e., minRxn/minFlux, see Fig. 1 and Methods).

We demonstrate the two-step procedure for three separate case studies of increasing complexity. The first one exhaustively identifies networks that convert glucose to acetate while conserving all carbon atoms with no provision for any additional co-reactants or co-products in the spirit of the recent study by Bogorad *et al.*^44^. The second study explores the reverse problem of identifying new ways of forming carbon-carbon bonds from the combined use of methanol and CO_2_ to stoichiometry-feasible C_2+_ products. Finally, the third study identifies suitable co-reactant and co-product pairs to drive forward the thermodynamically unfavorable methane to acetate conversion. The two-stage procedure recapitulates existing pathway designs^45^,^46^,^47^,^48^ and identifies novel pathway topologies inaccessible to existing algorithms. Overall conversion stoichiometries and pathway designs span a range of complex network topologies and provide valuable insight as to how the overall conversion pathway changes in response to alternate co-reactant and/or co-product scenarios.

## Results

### Brief description of the proposed two-step workflow

**Figure 1:**
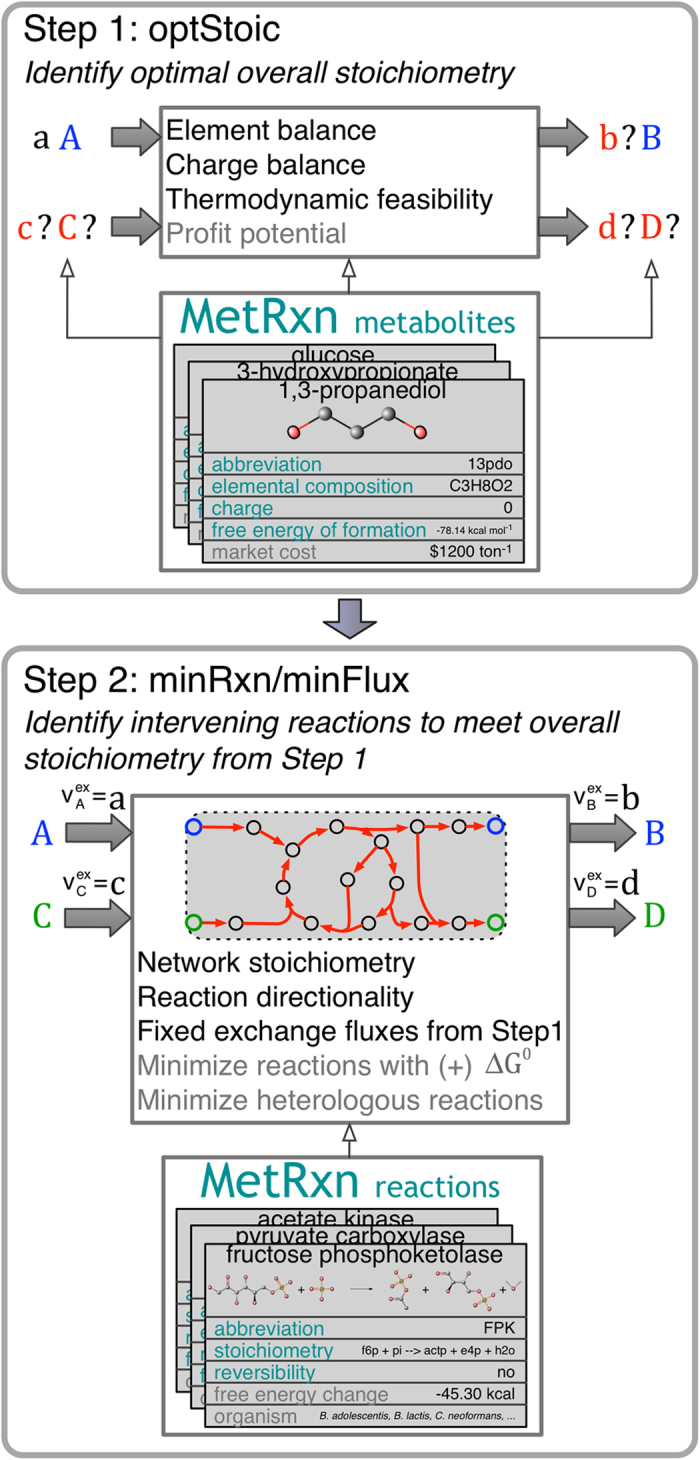
Pictorial representation of the two-step (optStoic + minRxn/minFlux) overall stoichiometry and reaction network design procedure. Step 1 (optStoic) identifies the overall stoichiometry of conversion from (**A** to **B**) maximizing a desired performance objective subject to overall constraints of elemental and charge balance and thermodynamic feasibility. Additional unknown co-reactant/co-product combinations (**C**, **D**) from a metabolite database and their respective stoichiometric coefficients in the overall conversion (**b**, **c**, **d**) are determined at this stage (in red). Step 2 identifies a network of reactions (in red) from a reaction database that meet the overall stoichiometry from Step 1 by either minimizing the number of reactions (minRxn) or the total metabolic flux (minFlux), subject to constraints of stoichiometric balance and reaction directionality. Additional constraints exploring alternate overall reaction stoichiometry (in Step 1) and network designs (in Step 2) are shown in grey.

The first step (optStoic) identifies the stoichiometric coefficients of the reactant and product metabolites in the overall conversion while maintaining thermodynamic feasibility considerations (Fig. 1). At this stage, not just the overall conversion stoichiometry but also some of the co-reactants and/or co-products are yet to be determined. Generally, the goal during this scoping phase is to identify an overall conversion that maximally utilizes a limiting carbon resource towards a target product while the intervening reaction steps are left at this stage unexplored. Ancillary considerations may include (i) co-utilization of other carbon substrates (e.g., gaseous reactants), (ii) possible co-production of valuable by-products, (iii) budgeting for a minimum level of biomass and growth or non-growth associated ATP, etc. Both the identity of some of the reactants and products as well as the overall stoichiometry are degrees of freedom that can be optimized towards meeting the above stated performance objectives. In addition to constraints on elemental and charge balances on the overall conversion respectively, a thermodynamic feasibility constraint is imposed to ensure that the free energy change of the overall conversion is below a pre-set negative threshold. Economic considerations implied by the prices of reactant and product molecules can also be imposed directly. The optStoic optimization formulation (see Methods for details) yields an LP model.

The second step searches through a database of elementally and charge balanced reactions (accessed from MetRxn^49^) to extract a (generally minimal) set of reactions that conform to the overall conversion stoichiometry identified under the first step (Fig. 1). Exchange reactions for only the metabolites present in the overall stoichiometry are added to allow for their uptake and export, respectively, and their exchange fluxes are fixed according to the overall stoichiometry determined by optStoic. Consequently, all flux from the reactant metabolites is routed directly or recycled through intermediates towards the target metabolites. The optimization problem could either minimize the total number of active reactions in the design (minRxn) or, alternately minimize the sum of non-zero fluxes (minFlux) to approximate the enzyme load required to achieve the overall conversion. Requirements on negativity of the free energy change of each individual reaction step, as well as number of heterologous reactions in the pathway can also be imposed to help rank-order all identified designs. minRxn yields an MILP optimization formulation whereas minFlux a much more tractable LP representation.

The two-stage stoichiometry and intervening reaction design procedure (optStoic + minRxn/minFlux) was implemented for three case studies that highlight the potential of the approach in identifying novel stoichiometries and network designs.

### Case Study 1: Synthetic pathways for fully converting glucose to acetate

Conversion of glucose through central carbon metabolism to acetyl-CoA (through EMP glycolysis or Entner-Doudoroff pathway) is required by most microorganisms for the production of biomass precursors and cofactors^50^. These pathways are also often recruited for the production of various bioproducts in a variety of production hosts. The generation of ATP and redox equivalent for growth, however, comes at the expense of losing two carbon atoms per glucose molecule as carbon dioxide reducing carbon yield to a maximum of 67%. Alternate modes of glucose metabolism, such as the glycerate pathway^51^ lead to the same carbon loss. An exception to this rule is found in some clostridial^52^ and lactate-producing bacteria^53^ that use a phosphoketolase enzyme to process glucose without any carbon loss to CO_2_. Recently, a synthetic, non-oxidative glycolysis (NOG) cycle was designed and assembled demonstrating the feasibility of this concept^44^ for bioconversion. By expressing in *E. coli* heterologous phosphoketolase enzymes xylulose-5-phosphate phosphoketolase (XPK) and fructose-6-phosphate phosphoketolase (FPK) from *B. adolescentis* Bogorad *et al.*^44^ assembled three cyclic networks demonstrating complete conversion of glucose to acetate. In this case study, we benchmark the proposed computational method for the same conversion and explore additional network designs beyond the ones described in Bogorad *et al.*^44^.

**Figure 2:**
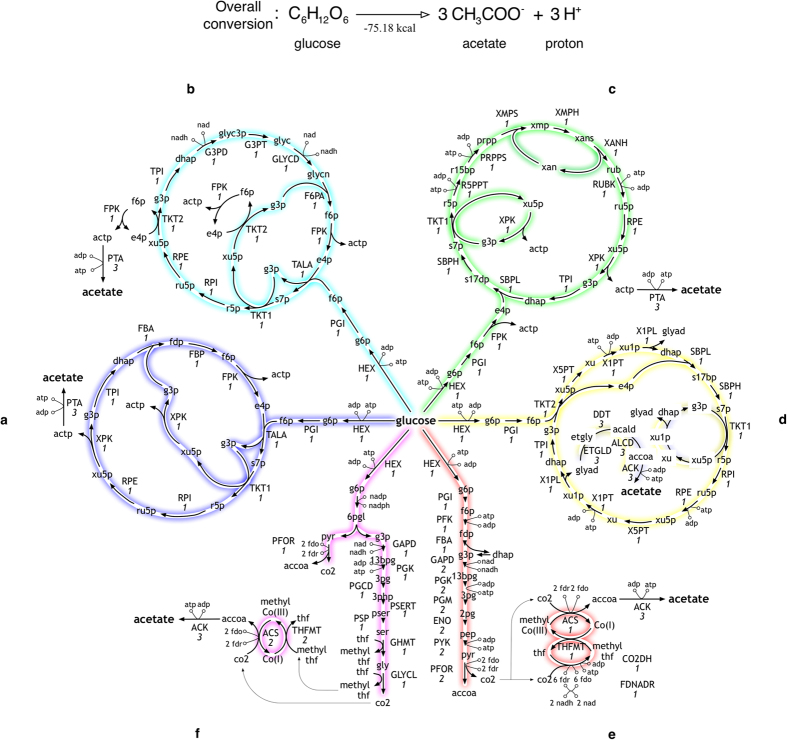
Six alternate network designs for conversion of glucose to acetate with 100% carbon efficiency. The full description of the abbreviated reaction (in upper case) and metabolite (in lower case) names is listed in Supplementary Data Files S3 and S4, respectively. The molar flux values for each reaction are shown in italics under the reaction names.

The first step (i.e., optStoic, see Methods) here is redundant as the overall stoichiometry in the form of *A* → 3*B* is fixed. Only networks that conserve all carbon flux from glucose to acetate satisfy the imposed overall stoichiometry. Results from both minRxn and minFlux (see Methods) converged to the same designs that achieve the overall conversion albeit with substantial differences in computational time. The smallest network (Fig. 2a) recapitulated the engineered construction of Bogorad *et al.*^44^ where glucose undergoes a stepwise conversion to acetyl phosphate (actp) in a cyclic route. A number of alternate routes using FPK or XPK as the sole deacetylating reaction were identified similar to those constructed in Bogorad *et al.*^44^ (see Fig. 2b for one of the constructions). Note that in this network design the need for cofactors NADH and NAD is balanced with a zero net requirement. minRxn/minFlux can also be used to directly limit the number of reactions with a positive standard change in free energy. While both NOG and all designs shown in Fig. 2a,b require at least one reaction with a positive standard free energy change (i.e., ribulose-5-phosphate isomerase (RPI)), the design depicted in Fig. 2c involves no reaction with positive Δ*G*^0^ (see Methods) whereby the thermodynamically unfavorable conversion of ribose-5-phosphate (r5p) to ribulose-5-phosphate (ru5p) is avoided by routing Pentose Phosphate Flux through C_5_ metabolism of phosphoribosyl pyrophosphate (prpp) and ribulose (rub) in *E. coli* (see Supplementary Text S1 for details). However, as a trade-off this cycle consumes one additional ATP compared to other designs. Networks not involving any phosphoketolase enzymes can also be designed (Fig. 2d) where glucose uses the modified RuMP pathway to produce three molecules of ethylene glycol. Diol dehydratase (DDT^54^) removes a water molecule from ethylene glycol to synthesize acetaldehyde (acald), which can be oxidized to acetate in three steps. This route can be potentially advantageous for the co-utilization of five and six carbon substrates^55^.

We next explored whether it is possible to construct the entire pathway in a single organism (see Methods) unlike the engineered NOG pathway that requires at least one heterologous enzyme (i.e., either FPK or XPK). Figure 2e illustrates one such design with reactions from only clostridial species such as *C. ljungdahlii*^56^ or thermophilic organisms such as *Moorella thermoacetica*^57^ (see Supplementary Data File S5). Here, the EMP pathway is combined with the Wood-Ljungdahl cycle to fix the CO_2_ lost in the conversion of pyruvate (pyr) to acetyl-CoA in pyr ferredoxin oxidoreductase enzyme (PFOR)^58^. Note that all network designs introduced here have a net zero ATP balance as specified in the overall stoichiometry. In contrast, the NOG pathway can produce two ATP molecules (per glucose molecule) from actp hydrolysis. By combining glycolysis and Wood-Ljungdahl we can in principle provide four ATP molecules (Fig. 2e). An alternate pathway (Fig. 2f) with a maximum generation of three ATP molecules can also be constructed using enzyme in the ED pathway. Here, the serine metabolism is utilized instead of the western branch of Wood-Ljungdahl cycle. However, practical hurdles in fixing all the CO_2_ released in pyruvate decarboxylation (PFOR) and glycine cleavage (GLYCL) may make these pathways difficult to engineer.

The trade-off in flux allocation between acetate production and cellular growth was assessed by including biomass formation (derived from the biomass equation in *i*AF1260 *E. coli* model^59^) as an additional product in the overall stoichiometry (see Methods). The study revealed that while maximum acetate production was limited by carbon availability at lower biomass levels, thermodynamic feasibility was the limiting factor at higher biomass levels due to increased demands for growth-associated maintenance ATP.

In this example, we examined how optimization formulations minRxn/minFlux can be used to select reactions that fully convert glucose to acetate while exploring the impact of minimizing reactions with a positive Δ*G*^0^, selection of production host and minimality of reactions in the designed conversion pathway.

### Case study 2: Co-utilization of methanol and carbon dioxide to C_2+_ compounds

In the second case study, we focus on the design of the overall conversion when not all participating metabolites are known *a priori* (see Methods). Methanol is an important commodity chemical and petrochemical by-product^60^ industrially produced from syngas. However, limitations in direct industrial catalytic condensation of methanol^61^ have so far limited the direct utilization of this low energy density molecule towards biofuel products such as ethanol and other C_2+_ molecules. A recent study has addressed this limitation by proposing and constructing a biological route for the direct condensation of methanol to ethanol^46^ with 100% efficiency of carbon conversion. Here we explore novel designs that can simultaneously convert methanol and co-utilize CO_2_ towards C_2+_ product metabolites with zero loss of carbon flux (i.e., *A* + *C* → *B* reaction). Note that product metabolite *B* is treated as unknown and could potentially be any of the 3,330 C_2+_ metabolites in set ***I*** (list of metabolites). The negative free energy change for methanol condensation (Δ*G* = −16.25 kcal mol^−1^) allows for the endergonic uptake of some CO_2_ to become thermodynamically feasible. As a result, designed pathways have the potential to achieve higher carbon efficiency than existing methanol condensation routes^46^ while also exhaustively exploring all possible C_2+_ product metabolites.

We chose maximization of the coefficient of CO_2_ in the overall stoichiometry as the design objective in optStoic while keeping the methanol coefficient at a value of one. This objective function maximizes the carbon yield of the desired product per mole of the limiting reactant (methanol). As many as 496 target metabolites from set ***I*** were identified as thermodynamically feasible products for the conversion (see Supplementary Data File S1 for full list). Table 1 shows ten of the designs in decreasing order of CO_2_ uptake. CO_2_ uptake varies in concert with the oxygen to hydrogen ratio (O:H) in the product metabolite. For example, malonate (C_3_H_4_O_4_), which has an O:H ratio of one, has the largest stoichiometric coefficient for CO_2_ (i.e., 1.25), while 2-methyl butanal (O:H = 0.1) has the lowest (i.e., 0.0714). As hydrogen availability in the reactants is fixed (stoichiometry of methanol is fixed to one), a low O:H ratio in the product reduces water production to satisfy elemental hydrogen balance. Because the oxygen atoms in CO_2_ are routed to water for products with low O:H ratio, reduced water production limits caused by the fixed availability of H, reduces the co-utilization of CO_2_ and thus overall carbon yield. It is important to note that the O:H ratio is also critical in deciding whether a desired metabolite is a feasible target product. For example, several platform chemicals such as ethylene glycol (O:H = 0.33), 1-butanol (O:H = 0.1) and 1,3-propanediol (O:H = 0.25) are not feasible solutions as mass conservation of hydrogen and oxygen cannot be simultaneously satisfied in the overall stoichiometry. Furthermore, metabolites with a very high O:H ratio, such as oxalate (O:H = 2) and glyoxylate (O:H = 1.5) are also not feasible due to limitations in oxygen atoms availability from CO_2_. Therefore, constraints of the overall design stoichiometry determine the feasibility of the target products as well as their maximum yields. It appears that in all designs thermodynamic feasibility is not the limiting constraint, instead elemental balances for H and O limit overall reaction stoichiometries.

**Figure 3:**
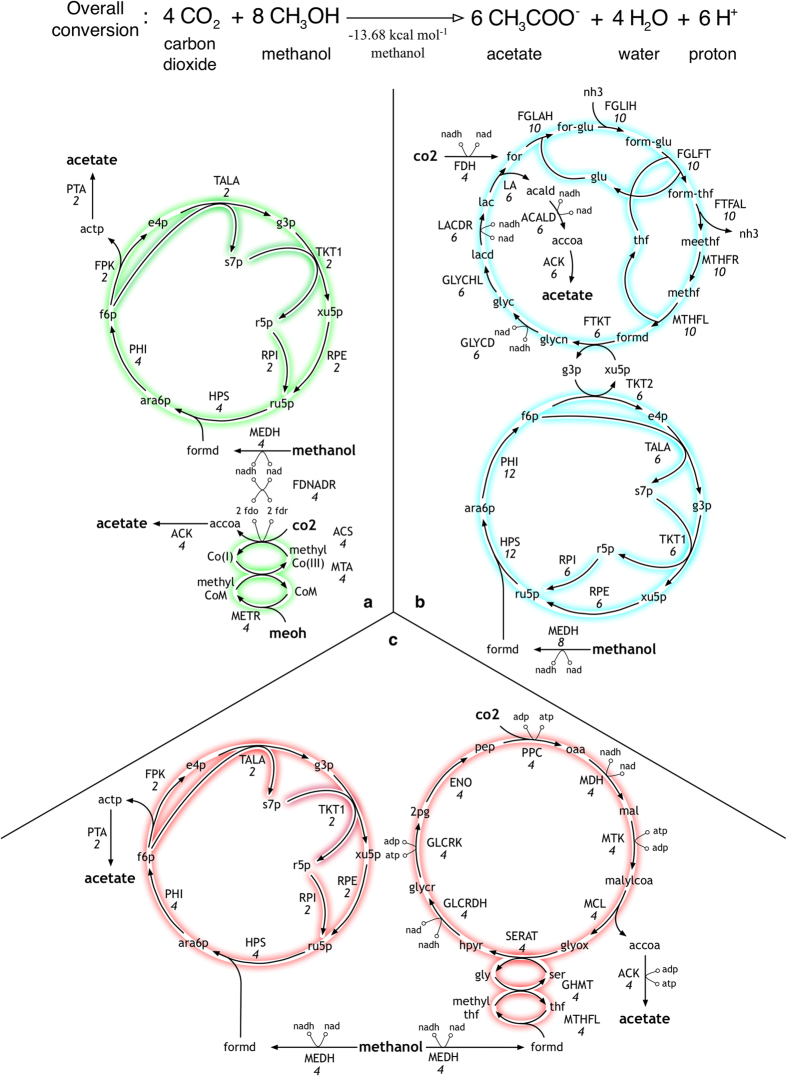
Network designs for the co-utilization of methanol and carbon dioxide towards acetate.

**Figure 4:**
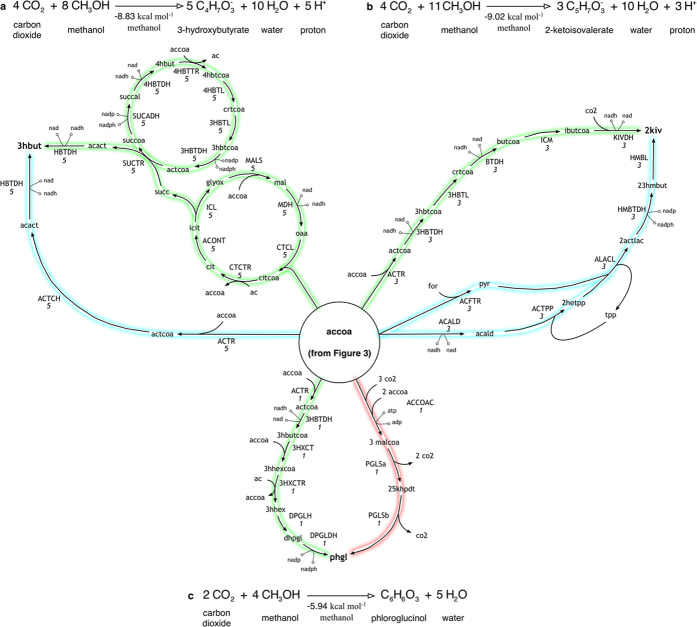
Designs for the co-utilization of methanol and carbon dioxide towards (**A**) 3-hydroxybutyrate (**B**) 2-ketoisovalerate and (**C**) phloroglucinol. The biosynthetic network for acetyl-CoA (accoa) from methanol and carbon dioxide is shown in Figure 3. Metabolic pathways explored in previous studies are highlighted in blue, while proposed ones are highlighted in green. Existing pathway for phloroglucinol production with lower energy efficiency is highlighted in red.

From Table 1, we selected four products (i.e., acetate, 3-hydroxybuyrate, 2-ketoisovalerate and phloroglucinol) for constructing networks satisfying their overall reaction stoichiometry (Figs 3 and 4). Both 3-hydroxybuyrate and 2-ketoisovalerate are platform chemicals^62^ that also serve as precursors to other biochemicals^41^,^62^ while phloroglucinol is a phenolic derivative used as a building block for cosmetics^63^ and explosives^64^. Note that for the construction of the networks we scaled up the overall stoichiometry such that all the coefficients are integers. Optimization formulations minRxn/minFlux revealed that the optimal reaction networks for each conversion involved a common core of reactions that initially convert the C_1_ substrates to an intermediate product (i.e., acetyl-CoA), which was subsequently routed towards the final product. As a result, the first example (acetate production) spans alternate routes of fixing CO_2_ with methanol to acetyl-CoA (and acetate). Each one of the identified networks is subsequently coupled with additional reactions to synthesize the three other target products (i.e., 3-hydroxybuyrate, 2-ketoisovalerate and phloroglucinol). The optimal network for converting methanol and CO_2_ to acetate is divided into two modules (Fig. 3a, Supplementary Text S1). The first module condenses four molecules of CO_2_ and methanol to synthesize four acetyl-CoA molecules using a combination of methylotropic and Wood-Ljungdahl pathway enzymes. The reducing equivalents (ferredoxin) required for powering this conversion is supplied by methanol oxidation in methanol dehydrogenase (MEDH) in the second module. This module assembles an alternative methanol condensation^46^ cycle where four formaldehyde molecules enter a modified ribulose monophosphate (RuMP) Pathway^65^ to condense to two actp molecules. Hexulose-6-phosphate synthase (HPS) and 6-phospho-3-hexulo isomerase (PHI) found in methylotrophs^65^ fix formd into the five-carbon ru5p backbone which is subsequently cleaved in FPK. Pentose Phosphate enzymes convert the intermediate substrates back to ru5p to complete the cycle using reactions also present in the NOG cycle.

The design shown in Fig. 3a requires the fewest number of reactions, however, the oxygen-sensitive nature of the Wood-Ljungdahl enzymes (MTA, ACS)^46^ could be a practical concern. The second synthetic design (Fig. 3b) uses histidine degradation metabolism^66^ and formate dehydrogenase (FDH) from clostridia^67^ to fix CO_2_ for acetate production. A third design using predominantly *E. coli* reactions (Supplementary Data File S5) was also identified (Fig. 3c). Here, half of the formaldehyde (from methanol) is fixed in the modified RuMP cycle and the rest is fixed along with CO_2_ in a functional reversal of the glyoxylate shunt^68^. As all the reactions in this design are either native to *E. coli* or have been successfully expressed in prior studies (malate thiokinase, malyl-CoA ligase)^68^, it has the fewest potential barriers for its implementation.

Each one of these cyclic routes can be combined with additional paths to route acetyl-CoA flux towards other target chemicals. Figure 4a–c show routes for converting methanol and CO_2_ to a C_4_, a C_5_ and a C_6_ product, respectively. Pathways for 3-hydroxybutyrate (3hbut) recapitulate existing engineered routes through acetoacetyl-CoA^69^,^70^ while also proposing additional synthetic designs that utilize succinate synthesis (through a modified TCA cycle) and metabolism (through *β*-oxidation reversal) routes (Fig. 4a). In addition to thiamine-dependent valine synthesis pathway for 2-ketoisovalerate (2kiv) production^71^, an alternate route linking *β*-oxidation reversal with valine degradation is also identified (Fig. 4b). In the case of phloroglucinol (phgl) production, however, the traditional pathway using malonyl-CoA metabolism^72^ was not identified by minFlux as the acetyl-CoA carboxylase (ACCOAC) step requires three additional ATP thus violating the overall energy balance fixed in optStoic (Fig. 4c in red). Instead, acetyl-CoA was converted to 3-hydroxybutyryl-CoA (3hbutcoa) which incorporates an additional acetyl-CoA molecule to form 3-hydroxy-5-oxohexanoyl-CoA (in 3-hydroxy-5-oxohexanoyl-CoA thiolase (3HXCT)). Cyclization and reduction of this molecule synthesizes phloroglucinol (Fig. 4c in green).

This case study highlights several of the advantages of the proposed procedure over existing pathway prospecting tools. Performance metrics can be directly imposed without the need for post-processing steps unlike existing procedures^31^,^41^,^73^. Constraints on overall stoichiometry filter out infeasible routes and unbalanced conversions. The designs are not restricted to linear paths^36^,^41^,^74^, but can explore cyclic routes connected not only by carbon transfer, but also through cofactor balances where the participating metabolites and their coefficients can be design variables.

### Case Study 3: Designing thermodynamically feasible paths for the conversion of methane to acetate

In the previous case study, only co-product (B) was unknown in the overall conversion. In the third case study, both co-reactant (C) and co-product (D) are design variables. With increased production of methane from natural gas deposits through advanced extraction technologies^75^, there is a growing interest of engineering metabolic routes of fixing this gaseous feedstock to C_2+_ liquid biofuel and biochemical targets^76^. However, the overall conversion creating a C-C bond by fixing methane with a C_1_ co-substrate (e.g., atmospheric CO_2_, CO from syngas, etc.) is thermodynamically unfavorable (e.g., Δ*G* = 11.05 kcal for *CH*_4_ + *CO*_2_ → *CH*_3_*COOH*). Recent studies have suggested that such a conversion can be achieved when coupled with a suitable electron acceptor^77^,^78^. Several anaerobic microorganisms have evolved ways to couple oxidation of methane with reduction of electron acceptors such as nitrite, sulfate^79^, iron and manganese^80^. In this study, we first identify all electron acceptors that could be used to convert methane to acetate (C_2_ product) and rank them according to their carbon yield to acetate. Subsequently, we select five solutions from the list of feasible electron acceptors to design pathways that convert methane and a C_1_ co-substrate (CO_2_ or CO) to acetate along with a C_2+_ platform chemical^1^,^81^.

Using optStoic and keeping the stoichiometry of acetate fixed at one (as a basis), we maximized the stoichiometric coefficient of methane while allowing the production of up to two additional metabolites from set ***I***. Non-carbon containing metabolites including metal ions, anions, protons and water were included as candidates in the overall stoichiometry. optStoic identified many distinct designs with alternative electron-acceptor combinations. Ten such designs are shown in Table 2 (see Supplementary Data File S2 for all the designs). Here, the overall reaction stoichiometry has been rescaled for unit molar consumption of the electron acceptor. The only feasible stoichiometry with a single additional co-metabolite was oxygen. We did not analyze further this stoichiometry as designs for aerobic conversion of methane have already been explored extensively^76^,^82^. Results demonstrate the varying capacity of different electron-accepting pairs in oxidizing methane to acetate due to thermodynamic considerations. The reduction of one mole of tetrathionate 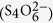 to hydrogen sulfide (H_2_S) accepts the maximum number of electrons (eighteen) thus converting 4.5 moles of methane to 2.25 moles of acetate. In contrast, one mole of iron(III)/iron(II) reduction 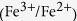 oxidizes only 0.25 moles of methane as it accepts only one electron. The electron acceptor pair 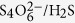 also supplies the oxygen required for the carbonyl group in acetate and oxidize additional hydrogen in methane to water. Most of the electron accepting pairs such as nitrate/nitrite 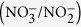 and bisulfite/hydrogen sulfide 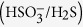 can perform both tasks. However, 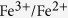 pair only performs the role of electron acceptor while water supplies the oxygen. Note that the commonly found electron pair sulfate/hydrogen sulfide reduction 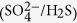 utilized by several marine anaerobic consortia for methane oxidation^83^,^84^ was not identified as a feasible solution as Δ*G*^*overall*^ of the reaction (−4.41 kcal mol^−1^ acetate) was below the conservatively chosen Δ*G^min^*(−5 kcal mole^−1^ acetate) for this case study.

For the second step we selected five pairs of electron acceptors from Table 2 (i.e., 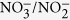, 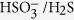, 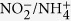, 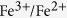 and 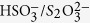) with different efficiencies of methane fixation per mole of oxidizing metabolite. For a unit mole conversion of each pair, we searched for a stoichiometry that further improves methane uptake by allowing for up to two additional metabolites from set ***I*** (similar to Step 1). The co-substrates were restricted to CO_2_ and CO while the co-products were chosen from all C_2+_ compounds (3,330 in set ***I***). Table 3 compares ten of the designs for 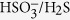 and 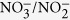 electron acceptors. Both acceptors exhibit the same general trend of higher methane utilization for products with a higher carbon ratio. In contrast to Case Study 2, it was the thermodynamic feasibility of the overall reaction and not the O:H ratio of the product that was limiting methane utilization. By allowing both the reactant and co-reactant stoichiometries to vary we have an additional degree to freedom to match the O:H ratio of the target product and maximize methane utilization all the way to the thermodynamic limit (i.e., Δ*G*^*overall*^ = −5 kcal). It is also interesting to note that 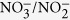
allows for much higher efficiency of methane fixation compared to 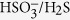. This is also because the thermodynamic driving force and not the electron-accepting capability of the electron acceptor becomes the limiting factor in this case.

**Figure 5:**
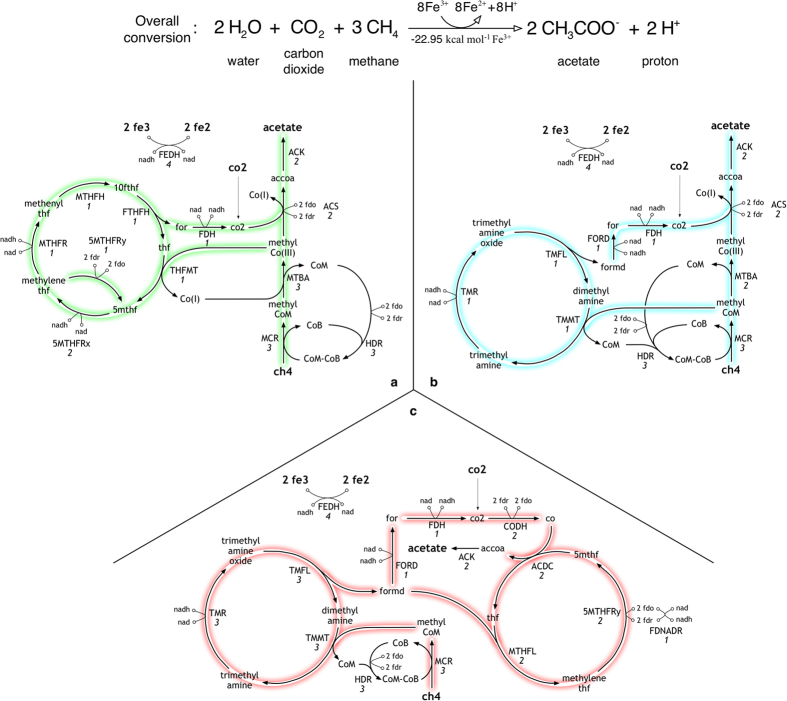
Network designs for fixing methane and carbon dioxide towards acetate using the Fe^3+^/Fe^2+^ electron acceptor pair.

**Figure 6:**
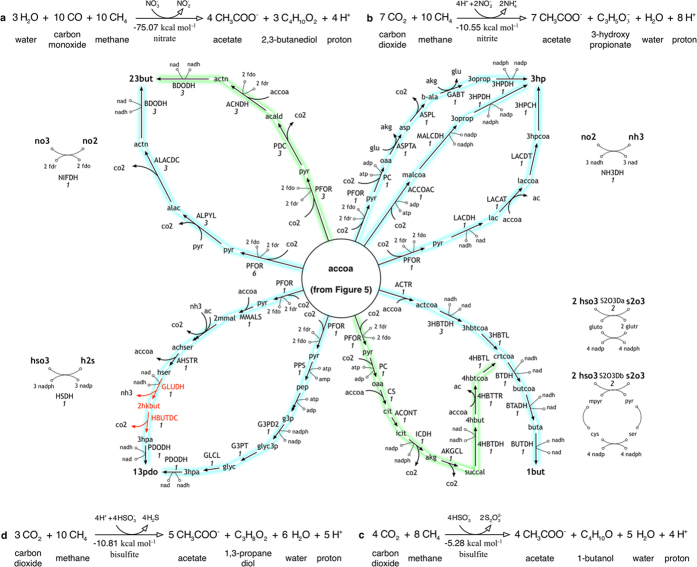
Network designs for conversion of methane and C_1_ co-reactant towards four C_2+_ target chemicals. Metabolic network of synthesis of acetyl-CoA (accoa) from methane (CH_4_) and co-metabolite (CO_2_ or CO) is shown in Figure 5. Metabolic pathways proposed in previous studies are highlighted in blue while new designs are drawn in green.

Each one of the five electron acceptors was used to suggest minimal networks for fixing methane to a different target product (see Figs 5 and 6). Similar to previous study, a core set of reactions first converted methane and the co-reactant to the intermediate acetyl-CoA. We chose the first alternative (i.e., 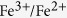) to explore ways of co-utilizing methane and CO_2_ (or CO) towards acetate (Fig. 5). Additional designs provide pathways from acetate to the final products (Fig. 6). All designs show that the terminal acceptors exchanged electrons with a cofactor pair instead of directly catalyzing a reaction in the carbon-transfer network. Different cofactors were identified for the electron acceptors depending on the reduction reactions in the network (see Supplementary Text S1 for details). Note that a few of the designs require electron transfer between NADH/NADPH and ferredoxin for regeneration of reduced ferredoxin (fdr) (e.g., see Fig. 5b,c). While the free energy change under standard conditions suggests that this reaction is favorable only in reverse (i.e., Δ*G*_*nad*/*fd*_ = 3.83 kcal^85^), there exists multiple experimental evidence alluding to the feasibility of electron transfer from NAD(P)H to ferredoxin^86^.

Figure 5 shows three alternate routes for the conversion of three molecules of methane and one molecule of CO_2_ to two acetate molecules with 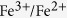 as the terminal electron acceptor. The smallest network (Fig. 5a) describes a functional reversal of the methanogenesis pathway where one molecule of methane and CO_2_ form acetyl-CoA using enzymes found in most methanogenic archaea and some methanotrophs^78^. However, in order to maintain the 1:1 ratio of methane and CO_2_ in the ACS reaction, one molecule of methane is oxidized to CO_2_ in a complex three-step cyclic route involving a functional reversal of the eastern branch of the Wood-Ljungdahl pathway (Fig. 5a). Alternate routes for oxidizing methane were also found that directly oxidize methyl-CoM to CO_2_ based on the methylamine cycle (Fig. 5b). The third design (Fig. 5c) bypasses the ACS and MTBA reactions by combining the two cycles described in Fig. 5a,b. Interestingly all the recruited enzymes (except for the terminal electron acceptors) are present in most anaerobic methanogens. This case study demonstrates how cofactor balances can become the dominant factor in synthetic pathway design. For example, the net reduction of 5-methyl tetrahydrofolate (5 mthf) to 5,10-methylene tetrahydrofolate is accomplished by two reactions running in opposite directions involving NAD and ferredoxin cofactors (Fig. 5a) to maintain overall balance of cofactors.

Figure 6 illustrates a few of the existing (in blue) and newly identified strategies (in green) for the production of four platform chemicals (i.e., 2,3-butanediol, 3-hydroxypropionate, 1-butanol and 1,3-propanediol^62^,^81^) each utilizing a different electron acceptor. For example, for 2,3-butanediol (23 but), while existing pathways (from pyruvate^87^) lose two carbon atoms for each molecule of 23 but, the identified pathways (Fig. 6a) preserve all carbon as lost CO_2_ is fixed back in the PFOR step. It is interesting to note that the identified pathway uses the same enzymes (except the MTA reaction) recruited for converting CO to 23 bdo in a recent study performed on three separate acetogenic clostridial species^48^ and functionally expressed in *E. coli*^88^. Three separate pathways were identified for 3-hydroxypropionate (3 hp) production all of which have been explored previously^47^,^89^,^90^ (Fig. 6b). Similarly, the shortest route for 1-butanol production (Fig. 6c) recapitulates existing strategies involving condensation of two molecules of acetyl-CoA by the accoa acetyltransferase (ACTR) followed by a functional reversal of the *β*-oxidation pathway^45^,^91^,^92^. A so far unexplored pathway is also suggested that combines the oxidative branch of TCA cycle with succinate-semialdehyde metabolism to reach 1-butanol (see Fig. 6c in green). For 1,3-propanediol (13 pdo) (Fig. 6d), the only identified pathway reduces acetyl-CoA back to glycerol, which is subsequently reduced to 13 pdo in three steps by the native reactions in some clostridial species^93^ or engineered enzymes found in *E. coli* and *K. pneumonie* (i.e., the DuPont pathway^94^). Our procedure could not identify an alternate pathway in *E. coli*^95^ where homoserine (hser) flux is routed towards 3-hydroxypropanal (3 hpa) by engineering the native glutamate dehydrogenase to be promiscuous towards homoserine. The reason is because the intermediate metabolite 4-hydroxy-2-oxobutyrate (4 hkbut) in the pathway as well as the reactions associated with the intermediate (see Fig. 6d in red) were absent in our database. Upon inclusion of the metabolite and the reactions, minFlux could identify this pathway as an alternate more energy efficient route for 13 pdo production.

In this study we identified and rank-ordered feasible electron acceptor pairs according to their methane oxidation potential. Unlike the previous example, thermodynamic feasibility and not elemental balances becomes the limiting factor in determining the optimum stoichiometric ratios in the overall reaction. minFlux identified relevant cofactor systems that coupled the methane utilization pathway with the terminal electron acceptors.

## Discussion

In this paper we introduced a two-step computational procedure (optStoic + minRxn/minFlux) for designing *de novo* synthetic networks that match an overall stoichiometry of chemical transformation. Performance criteria on the designed pathways (e.g., carbon yield/ energy efficiency, pathway Δ*G* etc.) can systematically be imposed to rank-order synthetic pathways according to the design objective. The case studies highlight the potential of this procedure for designing a thermodynamically feasible overall conversion stoichiometry, selecting the optimum combination of co-metabolites and co-products, and constructing non-intuitive synthetic biological routes for the overall conversion. We compared the optimal pathways identified here with two existing pathway design tools (Chou *et al.*^96^ and Bar-Even *et al.*^31^) for all three case studies. The results showed that the depth-first graph search algorithm for Chou *et al.*^96^ identified only linear paths connecting the primary substrate to the primary product for all the case studies, while failing to identify any of the carbon-conserving cyclic networks (Supplementary Text S1, Figure S6). The Bar-Even *et al.*^31^ approach successfully recapitulated the NOG cycle (Case Study 1), however, cofactor imbalances were introduced for the last two studies (Supplementary Text S1, Figure S6). The comparison demonstrates that designing first an overall stoichiometry enables homing in on only the designs with maximum carbon efficiency, particularly for conversions involving multiple substrates (or products).

Existing databases such as MetRxn^49^ or KEGG^97^ span a very large number of intermediate metabolites that can be produced from the reacting metabolites. In contrast, the number of terminal pathways towards target products tends to be much more limited. For example, acetyl-CoA participates in 153 reactions, whereas 13 pdo and phloroglucinol are involved in one and two reactions, respectively. Therefore, most of the novel network designs suggested by minRxn/minFlux stemmed from alternate intermediate metabolites and ways of producing them. Inclusion of additional metabolites and reactions (especially involved in the production/consumption of terminal metabolites) in the database could allow for the identification of additional conversion pathways (e.g., 13 pdo production in Case Study 3). Another promising way of expanding the diversity of designs could be to consider hypothetical biotransformations generated by successively applying reaction operators on metabolites^42^,^98^. For example, in a recent work for synthesizing platform chemicals in *E. coli*, GEM-Path^41^ suggested a pathway for 13 pdo production from malate that involved two *de novo* reactions. By combining minRxn/minFlux with compilations of hypothetical reactions and metabolites (e.g., BNICE^98^, GEM-Path^41^, Pertusi *et al.*^40^) the scope and diversity of designed synthetic networks would increase significantly. In addition, kinetic and toxicity information^99^,^100^ can be incorporated within minRxn/minFlux to further restrict the design space by avoiding kinetic bottlenecks and toxic intermediates. For example, maximum enzyme activity information could be used in addition to thermodynamics to set the bounds for the reaction fluxes.

## Methods

The detailed description of the two-step path-finding procedure is described in the following sections.

### Step 1: Determining optimal reactant and product combination and overall stoichiometry

The procedure begins with an optimization task that establishes the maximum achievable yields of the envisioned bioconversion subject to thermodynamic restrictions. This optimization task requires access to a database of metabolites along with specific data for each one of them. In this paper we assembled a database of 5,753 metabolites extracted from the MetRxn database^49^ (see Supplementary Data File S3). The following parameters are introduced that keep track of the elemental composition, charge information and free energy of formation data needed to describe the optimization problem:

*n*_*iq*_: # of atoms of element *q* in metabolite *i*

*e*_*i*_: Charge of metabolite *i*^101^


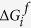
: Transformed free energy of formation (at 25 °C and 0.1 M ionic strength) of metabolite *i*^102^

where *i* = 1, …, N is the set ***I*** of metabolites present in the database and 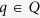 is the set of elements found in the metabolites (i.e., C, O, N, P, S, H, Fe, etc.) (see Supplementary Data File S3 for dataset).

For the sake of simplicity of presentation the optimization formulation is derived for the following bimolecular stoichiometry as shown in Fig. 1:


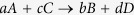


Using this representation either all the metabolites participating in the reaction are known *a priori* implying that all coefficients are positive and non-zero, or the presence of some of them is uncertain in which case the corresponding stoichiometric coefficients are allowed to assume a value of zero. Only one of the stoichiometric coefficients needs to be fixed to a finite value to provide a scaling for the remaining ones. Generally, we fix either the coefficient of the limiting carbon substrate or the primary product. By fixing this coefficient the presence of the corresponding reactant or product in the overall conversion is guaranteed. Note that setting the stoichiometric coefficient of a one-carbon molecule to one may not ensure that all other coefficients can assume integer values due to the balances on all other elements. However, all determined coefficients could be suitably scaled up to obtain an integer-only stoichiometry for all metabolites. The performance criteria of the overall conversion may involve (i) maximum yield for primary product B (i.e. max b while a = fixed), (ii) maximum co-utilization of free reactant C (max c, b = fixed) for a given product yield, (iii) minimum formation of by-product D (min d while b = fixed) or combinations thereof. The overall optimization formulation for Step 1 is as follows (optStoic):


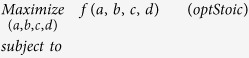



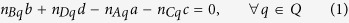



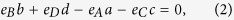



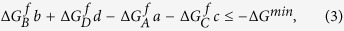



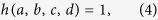



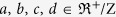


In formulation (optStoic), 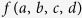 is a function of the overall stoichiometric coefficients quantifying a performance objective, Constraint (1) and (2) impose elemental and charge balances on the overall conversion respectively, while Constraint (3) imposes overall thermodynamic feasibility 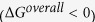. 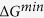 quantifies the amount of “extra” 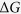 available for the designed stoichiometry. The stoichiometry of the basis metabolite (usually the limiting carbon source) is set to a value of one. Constraint (4) generalizes the description of this scaling decision. Stoichiometric coefficients *a*, *b*, *c*, *d* can be declared as integer or real depending on the adopted scaling of the overall conversion. It is straightforward to extend the optStoic formulation to account for more than two reactants and two products. In fact, a comprehensive list of putative co-reactants and co-products could be designed in the overall stoichiometry and then rely on the optStoic optimization problem to identify the optimal combination of co-reactants and co-products. Additional binary variables, associated with each metabolite from set ***I***, are included in the formulation to decide whether the respective metabolite should be a part of the overall stoichiometry or not. A negative value for a stoichiometric coefficient declares a metabolite as a reactant while a positive value defines it as a product. Here, both the participating metabolites and their respective coefficients are decision variables, and the modified optStoic formulation is a mixed integer linear programming (MILP) problem or an integer programming problem (IP) depending on whether the stoichiometric coefficients are declared as real or integer variables respectively.

The impact of cellular growth requirements on the flux allocation towards the target metabolite can be directly assessed by updating the overall stoichiometry to include biomass, growth (and non-growth) associated maintenance ATP as additional product metabolites:


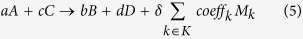


Here positive variable *δ* is the cellular growth coefficient, and biomass is represented as a weighted (by *coeff*_*k*_) sum of all the biomass precursors *M*_*k*_ (i.e., amino acids, lipids, cofactors as well as ATP requirements for cellular growth). Constraints for elemental and charge balances, and thermodynamic feasibility limitations are updated accordingly in optStoic:


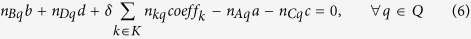



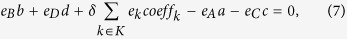



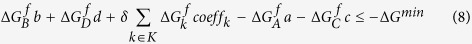


The trade-off between product yield and biomass formation can be assessed by fixing *δ* at different levels between zero and maximum growth and subsequently maximizing the target product coefficient.

Profit margin considerations (e.g., by using bulk market price of chemicals^103^) can also be imposed in the optStoic formulation by specifying that the product prices exceed the reactant costs by a pre-specified margin *profit*^*min*^.


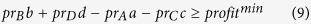


where *pr*_*i*_ are the per mole prices of reactants/products *i*. Alternatively this margin could become the objective function that is maximized if an economic criterion is used to select reactant/product pairs. Trade-offs between the overall conversion stoichiometry and the negativity of the overall free energy change can be drawn by varying the value of Δ*G*^*min*^. For large values of Δ*G*^*min*^ overall conversions are obtained with a high thermodynamic driving force. As Δ*G*^*min*^ is throttled back closer to zero more yield efficient conversions are obtained at the expense of a lower thermodynamic driving force that may limit flux per enzyme loading. Given that biomass formation is an endergonic process 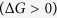 it is important to keep Δ*G*^*min*^ above the value needed for the desired growth. For example, for the *i*AF1260 metabolic model we find that the production of all constituents of biomass require a Δ*G* of 492.77 kcal g^−1^ biomass. Additional reserves for Δ*G* are needed to account for polymerization free energy costs of the biomass precursors not captured in the biomass equation. Alternatively, the overall stoichiometry could be expanded to directly include biomass, growth and non-growth associated maintenance ATP as product metabolites as described above.

### Step 2: Identifying reactions that conform to the identified overall stoichiometry

The second step identifies the smallest network of reactions that apportions the elemental composition of the substrates to the desired products in their corresponding stoichiometric ratios as determined in Step 1 (Fig. 1). To this end we extracted a set of mass and charge balanced reactions ***J*** (i.e., 6,871 reactions) from the MetRxn database^49^ involving metabolites from set ***I*** (see Supplementary Data File S4 for datasets) with additional exchange reactions for only the metabolites present in the overall stoichiometry to allow for their uptake and export, respectively. The optimization formulation for identifying the minimal number of reaction to match the identified stoichiometry requires the definition of the following parameters:

*S*_*ij*_: Stoichiometry matrix that describes the coefficient of metabolite *i* in reaction *j*


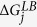
, 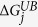: Lower and upper bound estimates for the Δ*G* of each reaction

(see Supplementary Data File S4 for dataset)

*LB*_*j*_, *UB*_*j*_: Lower and upper bounds on the flux of each reaction *j*

The change in free energy Δ*G* of a reaction is a function of the non-standard activities of the participating metabolites as follows:


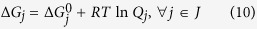


where *R* is the gas constant and *T* is the temperature. 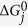 is the free energy change of the reaction under standard conditions. Note that *Q* is the reaction quotient expressed as the ratio of the product of the activities (approximated by their molar concentration in an aqueous solution) of the products raised to their stoichiometric coefficient to that of the reactants. The reaction quotient *Q* for the overall conversion *aA* + *cC* → *bB* + *dD* is:


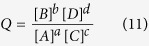


where the expressions within brackets are the molar concentrations of each participating metabolite. The minimum 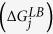 and maximum 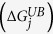 bounds on Δ*G* for each reaction in ***J*** are obtained by varying the concentration of the metabolites participating in the reaction by 1,000 fold of their transformed standard concentration (see Supplementary Data File S3). The wide range was chosen as a conservative estimate to allow for extreme fluctuations in intracellular metabolite concentrations in microorganisms (e.g., Bennet *et al.*^104^ reported intracellular metabolite ranges in exponentially growing *E. coli* to vary between 10^−3^–10^2^ mM). Reaction bounds *LB*_*j*_ and *UB*_*j*_ are determined according to the following rule:


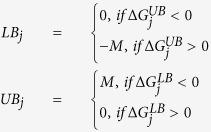


where *M* is a large positive value (i.e., *M* = *1,000*) in relation the exchange flux of the metabolites of the overall stoichiometry (from Step 1). Additional information on directionality of reactions obtained from literature can be used, whenever available, to further constrain *LB*_*j*_and *UB*_*j*_. The directionality for 4,447 reactions (out of 6,871 within set ***J***) was assigned by this procedure while the remaining 2,424 that lacked the free energy of formation for one or more metabolite were treated as reversible. Out of the 4,447 analyzed reactions, 2,205 were found to be irreversible whereas 2,242 were reversible. The identification of the minimal set of reactions that can match the desired overall stoichiometry (found after Step 1) is formulated as the following MILP (minRxn) problem:


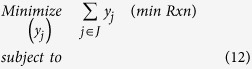



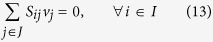



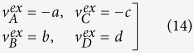



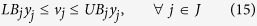



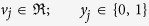


Binary variables *y*_*j*_ defined as


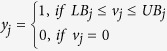


control the addition (or not) of reaction *j* in the overall network. The objective function involves the minimization of the sum of *y*_*j*_’s (Constraint 12) to extract the minimum number of reactions that can support the desired overall stoichiometry (optStoic) of Step 1. Constraint (13) ensures that all metabolites, including cofactors, are used in a stoichiometrically balanced manner. Constraint (14) sets the flux of the exchange reactions (i.e., for A, B, C and D) equal to their respective stoichiometric coefficients thus matching the overall desired stoichiometry (from Step 1) and providing a flux scaling. Constraint (15) ensures that reaction *j* is removed from the designed network when *y*_*j*_ is equal to zero. Networks for different overall stoichiometries (from Step 1) can be obtained and contrasted for their overall performance metrics. The use of integer cut constraints to (i) disallow previously identified solutions and (ii) search for alternate optimal as well as sub-optimal solutions can be used to exhaustively explore all possible ways of linking reactants to products (Constraint 16).


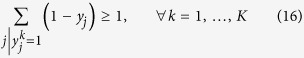


where *k* = 1, …, *K* is the set previously of explored solutions.

The resulting (minRxn) MILP formulation can be computationally challenging to solve as it involves as many as 6,871 binary variables associated with all the reactions in set ***J***. A related LP formulation (minFlux) that is significantly (i.e., up to three orders of magnitude) less computationally taxing can be solved as a surrogate of minRxn. In contrast to minRxn that directly minimizes the total number of reactions in the designed network, minFlux minimizes the total metabolic flux of the chosen reactions. This modified objective function could be viewed as an approximation of the total enzyme-load imposed on the organism^105^:


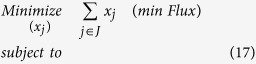



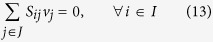



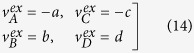



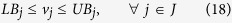



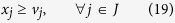



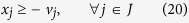



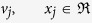


In minFlux, the sum of the absolute values of the reaction fluxes denoted by *x*_*j*_ is minimized (Constraint 17). Constraints (13) and (14) are the same as in (minRxn) formulation whereas Constraint (18) imposes bounds on fluxes without multiplication with a binary variable. Constraints (19) and (20) ensure that *x*_*j*_ is greater than both *v*_*j*_ and −*v*_*j*_. The minimization operator in the objective function ensures that for every *j* either Constraint (19) or (20) becomes active.

Alternate optimal (as well as suboptimal) basic flux combinations can also be explored by preventing all nonzero basic variables from an earlier solution *k* from reappearing in subsequent flux combinations (see Lee *et al.*^106^ for details). In short, we first reorganize minFlux in standard form^107^ and transform all the variables (i.e., *x*_*j*_ and *v*_*j*_) to positive variable *s*_*l*_ (where ***L*** is the set of variables for problem in standard form). Subsequently, a set of integer cut constraints similar to the ones described for formulation (minRxn) can be constructed. Binary variables *u*_*l*_ denote the presence of nonzero basic variable *l* in a previously identified solution *k*. The following constraints prevents the reappearance of a previously found basic feasible solution:


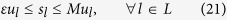



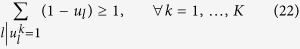


where 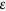 is a small positive value 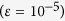 that ensures that *s*_*l*_ is nonzero when *u*_*l*_ = 1. It is important to stress that the addition of integer cuts converts the LP (minFlux) formulation to an MILP problem. Nevertheless, it is can be solved significantly faster (about two orders of magnitude) than minRxn as it involves much fewer binary variables.

Additional constraints can be added in either minRxn or minFlux optimization formulations to maintain the total number of reactions with a positive standard change in free energy at a minimum (e.g., less than *p*^*t*^):


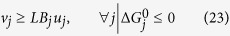



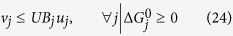



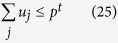


where *u*_*j*_ is a binary variable which is defined as:


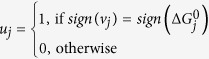


Reactions with a positive standard change in free energy may require the accumulation of a significant reactant metabolite pool to drive the reaction forward. This is generally undesirable as it may throttle back the flux in the overall pathway by triggering cellular toxicity and/or pathway substrate inhibition.

In addition, formulations minRxn or minFlux can be augmented to control the number of organisms from which reactions are sourced in the construction of the conversion network to less than *p*^*o*^.


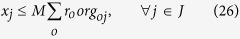



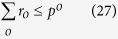



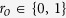


Here, parameter *org*_*oj*_ has a value of one if reaction *j* is present in organism *o*, and zero otherwise. Using binary variable *r*_*o*_, Constraint (26) ensures that a reaction *j* cannot carry any flux unless it is present in at least one organism *o* (i.e., 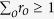). Constraint (27) sets an upper bound *p*^*o*^ on the number of organisms contributing reactions. One can avoid selecting the host organism *a priori,* thus allowing the optimization formulation to select the production host(s) best suited to construct the synthetic network. In addition, the definition of set ***O*** can be altered accordingly to minimize diversity in chosen genus instead of individual organisms. Alternatively, the production host could be pre-specified and then restrict the total number of heterologous reactions.

## Additional Information

**How to cite this article**: Chowdhury, A. and Maranas, C. D. Designing overall stoichiometric conversions and intervening metabolic reactions. *Sci. Rep.* **5**, 16009; 10.1038/srep16009 (2015).

## Supplementary Material

Supplementary Text S1

Supplementary Data File S1

Supplementary Data File S2

Supplementary Data File S3

Supplementary Data File S4

Supplementary Data File S5

## Figures and Tables

**Table 1:** Overall stoichiometry designs (with stoichiometric coefficients rounded off to two decimal places) for the conversion of methanol and CO_2_ to ten C_2+_ metabolites.

Product	Stoichiometry	O:H ratio
Malonate	1.25 CO_2_ + CH_3_OH 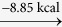 0.75 C_3_H_4_O_4_ + 0.5 H_2_O	1.00
Citrate	1.00 CO_2_ + CH_3_OH 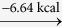 0.33 C_6_H_8_O_7_ + 0.67 H_2_O	0.88
Succinate	0.71 CO_2_ + CH_3_OH 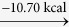 0.43 C_4_H_6_O_4_ + 0.71 H_2_O	0.67
Acetate	0.50 CO_2_ + CH_3_OH 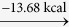 0.75 C_2_H_4_O_2_ + 0.5 H_2_O	0.50
Phloroglucinol	0.50 CO_2_ + CH_3_OH 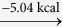 0.25 C_6_H_6_O_3_ + 1.25 H_2_O	0.50
3-Hydroxypropionate	0.50 CO_2_ + CH_3_OH 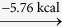 0.50 C_3_H_6_O_3_ + 0.5 H_2_O	0.50
2-Ketoisovalerate	0.36 CO_2_ + CH_3_OH 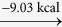 0.27 C_5_H_8_O_3_ + 0.91 H_2_O	0.38
3-Hydroxybutyrate	0.33 CO_2_ + CH_3_OH 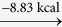 0.33 C_4_H_8_O_3_ + 0.67 H_2_O	0.38
2,3-Butanediol	0.09 CO_2_ + CH_3_OH 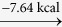 0.27 C_4_H_10_O_2_ + 0.64 H_2_O	0.20
2-Methylbutanal	0.07 CO_2_ + CH_3_OH 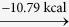 0.21 C_5_H_10_O + 0.93 H_2_O	0.10

The overall stoichiometries maximize CO_2_ uptake for unit molar uptake of methanol. The oxygen-to-hydrogen ratio of the target metabolites is shown in the rightmost column. The free energy change of reaction is shown above the reaction stoichiometry.

**Table 2:** Ten feasible electron acceptors for driving conversion of methane (CH_4_) to acetate (CH_3_COO^−^).

Electron acceptor	Stoichiometry
Tetrathionate/Hydrogen Sulfide	4.5 CH_4_ + 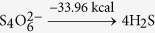 + 2.25 CH_3_COO^−^ + 0.25 H^+^ + 1.5 H_2_O
Nitrate/Ammonium	2 CH_4_ + 1 H^+^ + 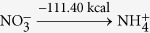 + 1 CH_3_COO^−^ + 1 H_2_O
Thiosulfate/Hydrogen Sulfide	2 CH_4_ + 2 H^+^ + 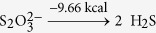 + 1 CH_3_COO^−^ + 1 H_2_O
Bisulfite/Hydrogen Sulfide	1.5 CH_4_ + 0.25 H^+^ + 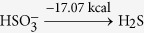 + 0.75 CH_3_COO^−^ + 1.5 H_2_O
Nitrite/Ammonium	1.5 CH_4_ + 1.25 H^+^ + 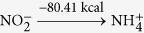 + 0.75 CH_3_COO^−^ + 0.5 H_2_O
Nitrous Oxide/Hydroxylamine	2.25 H_2_O + 1.25 CH_4_ + 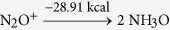 + 0.625 CH_3_COO^−^ + 1.625 H^+^
Nitrate/Nitrite	0.5 CH_4_ + 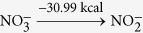 + 0.25 CH_3_COO^−^ + 0.25 H^+^ + 0.5 H_2_O
Bisulfite/Thiosulfate	0.5 CH_4_ + 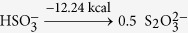 + 0.25 CH_3_COO^−^ + 0.25 H^+^ + 1 H_2_O
Sulfur dioxide/Tetrathionate	0.375 CH_4_ + 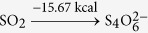 + 0.1875 CH_3_COO^−^ + 0.6875 H^+^ + 1 H_2_O
Iron(III)/Iron(II)	0.25 H_2_O + 0.25 CH_4_ + 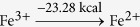 + 0.125 CH_3_COO^−^ + 1.125 H^+^

The overall stoichiometries maximize methane uptake for unit molar conversion of each electron acceptor. The free energy change of reaction is shown above the reaction.

**Table 3:** Limiting overall stoichiometries for acceptor pairs 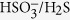 and 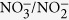 for ten target products.

Product	Stoichiometry 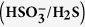	Stoichiometry 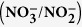
Isobutanoate	5.62 CH_4_ + 2.57 CO_2_ 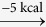 C_2_H_4_O_2_ + 1.55 C_4_H_8_O_2_	9.40 CH_4_ + 5.64 CO_2_ 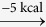 C_2_H_4_O_2_ + 3.26 C_4_H_8_O_2_
Succinate	3.48 CH_4_ + 2.47 CO_2_ 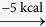 C_2_H_4_O_2_ + 0.99 C_4_H_6_O_2_	4.88 CH_4_ + 5.41 CO_2_ 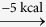 C_2_H_4_O_2_ + 2.07 C_4_H_6_O_2_
1-Butanol	3.47 CH_4_ + 0.82 CO_2_ 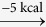 C_2_H_4_O_2_ + 0.57 C_4_H_10_O	4.87 CH_4_ + 1.96 CO_2_ 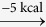 C_2_H_4_O_2_ + 1.21 C_4_H_10_O
Acetone	3.44 CH_4_ + 1.10 CO_2_ 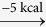 C_2_H_4_O_2_ + 0.85 C_3_H_6_O	4.82 CH_4_ + 2.53 CO_2_ 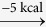 C_2_H_4_O_2_ + 1.78 C_3_H_6_O
Isopropanol	3.40 CH_4_ + 0.80 CO_2_ 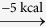 C_2_H_4_O_2_ + 0.73 C_3_H_8_O	4.71 CH_4_ + 1.90 CO_2_ 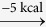 C_2_H_4_O_2_ + 1.54 C_3_H_8_O
1-Propanol	3.23 CH_4_ + 0.74 CO_2_ 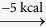 C_2_H_4_O_2_ + 0.66 C_3_H_8_O	4.35 CH_4_ + 1.78 CO_2_ 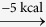 C_2_H_4_O_2_ + 1.38 C_3_H_8_O
2,3-Butanediol	2.82 CH_4_ + 0.74 CO_2_ 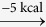 C_2_H_4_O_2_ + 0.39 C_4_H_10_O_2_	3.50 CH_4_ + 1.77 CO_2_ 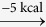 C_2_H_4_O_2_ + 0.82 C_4_H_10_O_2_
3-Hydroxypropionate	2.61 CH_4_ + 1.11 CO_2_ 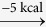 C_2_H_4_O_2_ + 0.58 C_3_H_6_O_3_	3.07 CH_4_ + 2.57 CO_2_ 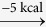 C_2_H_4_O_2_ + 1.21 C_3_H_6_O_3_
1,3-Propanediol	2.50 CH_4_ + 0.62 CO_2_ 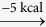 C_2_H_4_O_2_ + 0.37 C_3_H_8_O_2_	2.82 CH_4_ + 1.54 CO_2_ 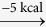 C_2_H_4_O_2_ + 0.79 C_3_H_8_O_2_
Ethylene glycol	2.23 CH_4_ + 0.56 CO_2_ 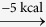 C_2_H_4_O_2_ + 0.41 C_2_H_8_O_2_	2.32 CH_4_ + 1.39 CO_2_ 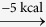 C_2_H_4_O_2_ + 0.86 C_2_H_8_O_2_

All the overall stoichiometries maximize methane uptake for unit molar conversion of each electron acceptor and unit molar production of acetate.
