# Supplementary material for: Designing overall stoichiometric conversions and intervening metabolic reactions
Source: Sci Rep. 2015 Nov 4;5:16009. doi: 10.1038/srep16009 (PMC4632160; doi:10.1038/srep16009)
Supplement: Supplementary Text S1 [file srep16009-s1.pdf]

Supplementary Text S1

Designing overall stoichiometric conversions and intervening metabolic reactions

Anupam Chowdhury[1] and Costas D. Maranas[1*]

[1]Department of Chemical Engineering, The Pennsylvania State University, University Park, PA 16802

[*]Corresponding author

E-mail: costas@psu.edu

Phone: 814-863-9958

Detailed description of the identified pathways identified by optStoic+minRxn/minFlux for the three case studies

<u>Case Study 1: Synthetic pathways for fully converting glucose to acetate</u>

The smallest design (Figure S1a) recapitulated the engineered construction of Bogorad *et al* [1] where glucose undergoes a stepwise conversion to acetyl phosphate (actp) in a cyclic route. A number of alternate routes using FPK or XPK as the sole deacetylating reaction were identified similar to those constructed in Bogorad *et al* [1] (see Figure S1b for one of the constructions). Note that in this network design the need for cofactors nadh and nad is balanced with a zero net requirement. minRxn/minFlux can also be used to directly limit the number of reactions with a positive standard free energy of change. While both NOG and all designs shown in Figure S1a and b require at least one reaction with a positive standard free energy change (i.e., ribulose-5-phosphate isomerase (RPI)), the design depicted in Figure S1c involves no reaction with positive ΔG^0 (see Methods) whereby the thermodynamically unfavorable conversion of ribose-5-phosphate (r5p) to ribulose-5-phosphate (ru5p) is avoided by routing Pentose Phosphate Flux through C_5 metabolism of phosphoribosyl pyrophosphate (prpp) and ribulose (rub) in *E. coli*. However, as a trade-off this cycle consumes one additional ATP compared to other designs. Networks not involving any phosphoketolase enzymes can also be designed (see Figure S1d) where glucose uses the modified RuMP pathway to produce three molecules of ethylene glycol. Diol dehydratase (DDT [2]) removes a water molecule from ethylene glycol to synthesize acetaldehyde (acald), which can be oxidized to acetate in three steps. This route can be potentially advantageous for the co-utilization of five and six carbon substrates [3].

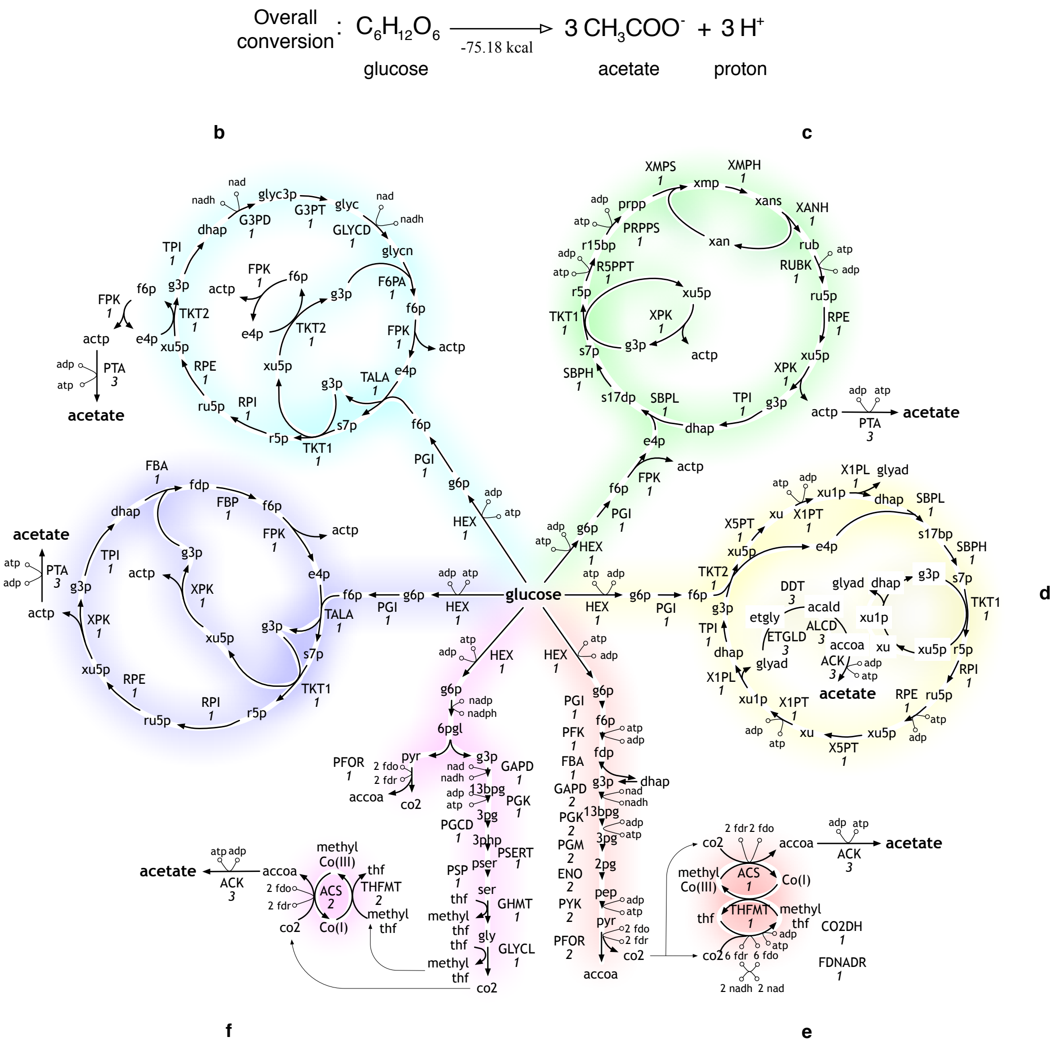

Figure S1: Six alternate network designs for conversion of glucose to acetate with 100% carbon efficiency. The full description of the abbreviated reaction (in upper case) and metabolite (in lower case) names is listed in Supplementary Data Files S3 and S4, respectively. The molar flux values for each reaction are shown in italics under the reaction names.

We next explored whether it is possible to construct the entire pathway in a single organism (see Methods) unlike the engineered NOG pathway that requires at least one heterologous enzyme (i.e., either FPK or XPK). Figure S1e illustrates one such design with reactions from only clostridial species such as *C. ljungdahlii* [4] or thermophilic organisms such as *Moorella thermoacetica* [5]. Here, the EMP pathway is combined with the Wood-Ljungdahl cycle to fix the CO_2 lost in the conversion of pyruvate (pyr) to acetyl-CoA. Two pyr molecules (from one glucose molecule) are decarboxylated by the pyr ferredoxin oxidoreductase enzyme (PFOR) [6] to acetyl-CoA and CO_2. Subsequently, one CO_2 molecule enters the western branch of Wood-Ljungdahl

cycle where it is reduced and fixed with tetrahydrofolate (thf) to 5-methyl tetrahydrofolate (methf). Next, methf enters the eastern branch of Wood-Ljungdahl pathway to condense with the second CO_2 molecule to synthesize the third molecule of acetyl-CoA. Conversion of acetyl-CoA to acetate completes the pathway. Note that all network designs introduced here have a net zero ATP balance as specified in the overall stoichiometry. In contrast, the NOG pathway can produce two ATP molecules (per glucose molecule) from actp hydrolysis. By combining glycolysis and Wood-Ljungdahl we can in principle provide four ATP molecules (see Figure S1e). An alternate pathway (see Figure S1f) with a maximum generation of three ATP molecules can also be constructed using enzyme in the ED pathway. Here, the serine metabolism is utilized instead of the western branch to generate methf. However, practical hurdles in fixing all the CO_2 released in pyruvate decarboxylation (PFOR) and glycine cleavage (GLYCL) may make these pathways difficult to engineer.

Case study 2: Co-utilization of methanol and carbon dioxide to C_{2+} compounds

Optimization formulations minRxn/minFlux revealed that the optimal reaction networks for each conversion involved a common core of reactions that initially convert the C_1 substrates to an intermediate product (i.e., acetyl-CoA), which was subsequently routed towards the final product. As a result, the first example (acetate production) spans alternate routes of fixing CO_2 with methanol to acetyl-CoA (and acetate). Each one of the identified networks is subsequently coupled with additional reactions to synthesize the three other target products (i.e., 3-hydroxybuyrate, 2-ketoisovalerate and phloroglucinol). The optimal network for converting methanol and CO_2 to acetate is divided into two modules (see Figure S2a). The first module condenses four molecules of CO_2 and methanol to synthesize four acetyl-CoA molecules using a combination of methylotropic and Wood-Ljungdahl pathway enzymes. The methyl group of methanol is transferred to Corrinoid protein by CoM-Corrinoid methyltransferases (MTA) [7,8], which subsequently condenses with CO_2 to acetyl-CoA by acetyl-CoA synthase (ACS) enzyme from the Wood-Ljungdahl pathway [9]. The reducing equivalents (ferredoxin) required for powering this conversion is supplied by methanol oxidation in methanol dehydrogenase (MEDH) in the second module. This module assembles an alternative methanol condensation [10] cycle where four formaldehyde molecules enter a modified ribulose monophosphate (RuMP) Pathway [11] to condense to two actp molecules. Hexulose-6-phosphate synthase (HPS) and 6-phospho-3-hexulo isomerase (PHI) found in methylotrophs [11] fix formd into the five-carbon ru5p backbone which is subsequently cleaved in FPK. Pentose Phosphate enzymes convert the intermediate substrates back to ru5p to complete the cycle using reactions also present in the NOG cycle.

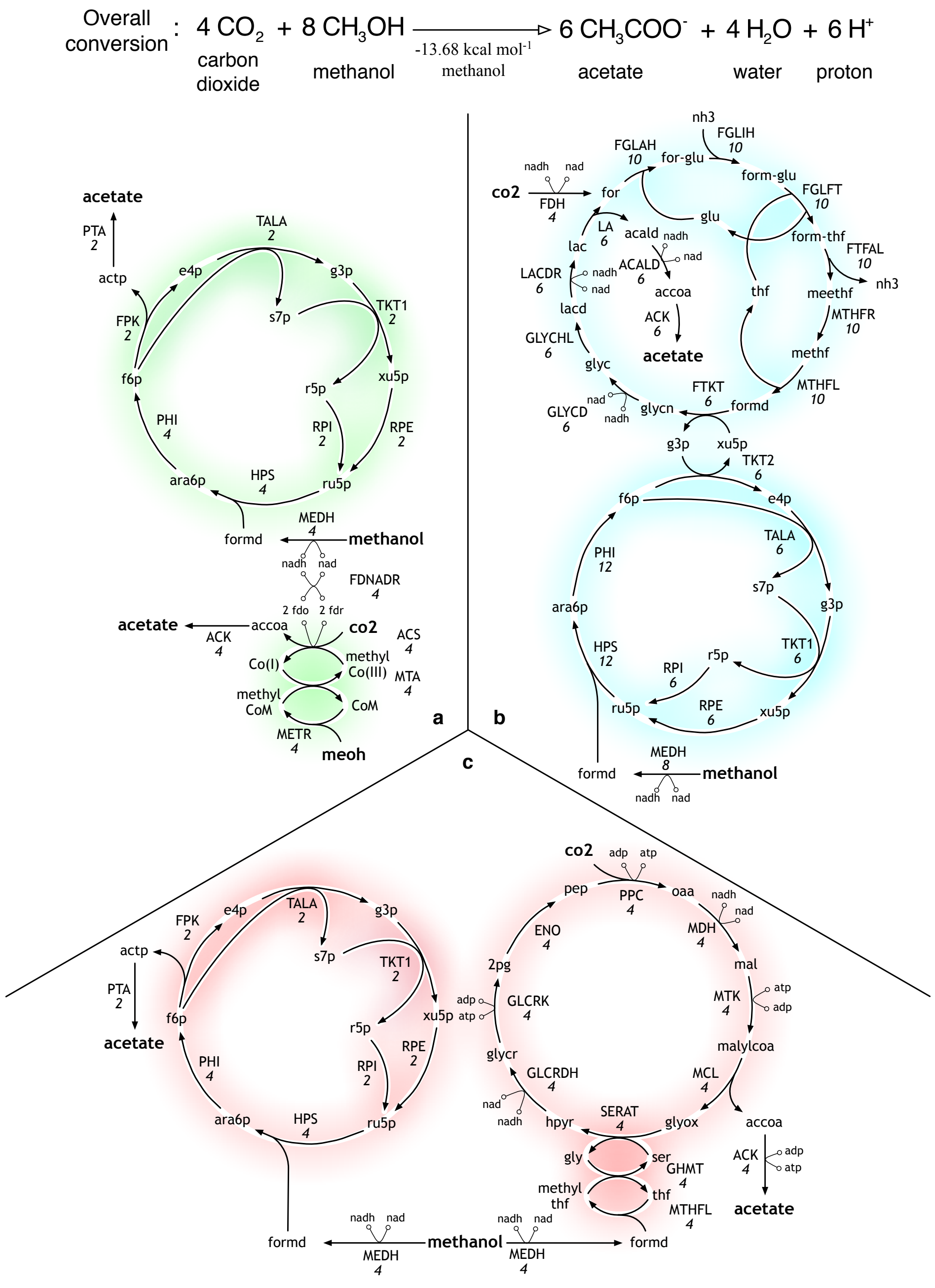

Figure S2: Network designs for the co-utilization of methanol and carbon dioxide towards acetate.

The design shown in Figure S2a requires the fewest number of reactions, however, the oxygen-sensitive nature of the Wood-Ljungdahl enzymes (MTA, ACS) [10] could be a practical concern. The second synthetic design (see Figure S2b) uses histidine degradation metabolism [12] and formate dehydrogenase (FDH) from *Clostridia* [13] to fix CO_2 for acetate production. Here, the RuMP cycle is altered to convert formaldehyde to glycerone instead of actp. Formaldehyde

transketolase (FTKT) from methylotropic yeasts such as *P. pastoris* [14] is the only additional reaction required to fix an additional formaldehyde molecule with xu5p, and subsequently cleave it to glycerone. Subsequently, glycerone follows lactate metabolism to acetaldehyde (acald) (in Lactate Aldolase (LA)), and oxidation of acetaldehyde completes the network.

A third design using predominantly *E. coli* reactions was also identified (Figure S2c). Here, half of the formaldehyde (from methanol) is fixed in the modified RuMP cycle and the rest is fixed along with CO_2 in a functional reversal of the glyoxylate shunt [15]. C_1 metabolism in the serine pathway is combined with serine-glyoxylate aminotransferase (SERAT) [16] to fix the remaining formaldehyde to glyoxylate and produce hydroxypyruvate (hpyr). Phosphoenolpyruvate (pep), from the reduction of hpyr in glycerate metabolism, fixes atmospheric CO_2 in the anaplerotic pep carboxylase enzyme (PPC). This flux is routed through malyl-CoA (in ATP-driven malate thiokinase (MTK) [15]) producing acetyl-CoA and glyoxylate through the malyl-CoA ligase (MCL). As all the reactions in this design are either native to *E. coli* or have been successfully expressed in prior studies (MTK, MCL) [15], it has the fewest potential barriers for its implementation.

Each one of these cyclic routes can be combined with additional paths to route acetyl-CoA flux towards other target chemicals. Figure S3a, b and c show routes for converting methanol and CO_2 to a C_4, a C_5 and a C_6 product, respectively. Pathways for 3-hydroxybutyrate (3hbut) recapitulate existing engineered routes through acetoacetyl-CoA [17,18] while also proposing additional synthetic designs that utilize succinate synthesis (through a modified TCA cycle) and metabolism (through β-oxidation reversal) routes (see Figure S3a). In addition to thiamine-dependent valine synthesis for 2-ketoisovalerate (2kiv) production [19], an alternate route linking β-oxidation reversal with valine degradation is also identified (see Figure S3b). Here, n-butanoyl-CoA in the C_4 β-oxidation cycle is isomerized to isobutyryl-CoA (ibutcoa) in iso-butyryl-CoA mutase (ICM) [20]. Reversal of 2-ketoisovalerate oxidoreductase (KIVDH) in the valine degradation pathway converts ibutcoa to the target product. In the case of phloroglucinol (phgl) production, however, the traditional pathway using malonyl-CoA metabolism [21] was not identified by minFlux as the acetyl-CoA carboxylase (ACCOAC) step requires three additional ATP thus violating the overall energy balance fixed in optStoic. Instead, acetyl-CoA was converted to hydroxyl-butyryl-CoA (hbutcoa) which incorporates an additional acetyl-CoA molecule to form 3-hydroxy-5-oxohexanoyl-CoA (in 3-hydroxy-5-oxohexanoyl-CoA thiolase (3HXCT)). Cyclization and reduction of this molecule synthesizes phloroglucinol (Figure S3c).

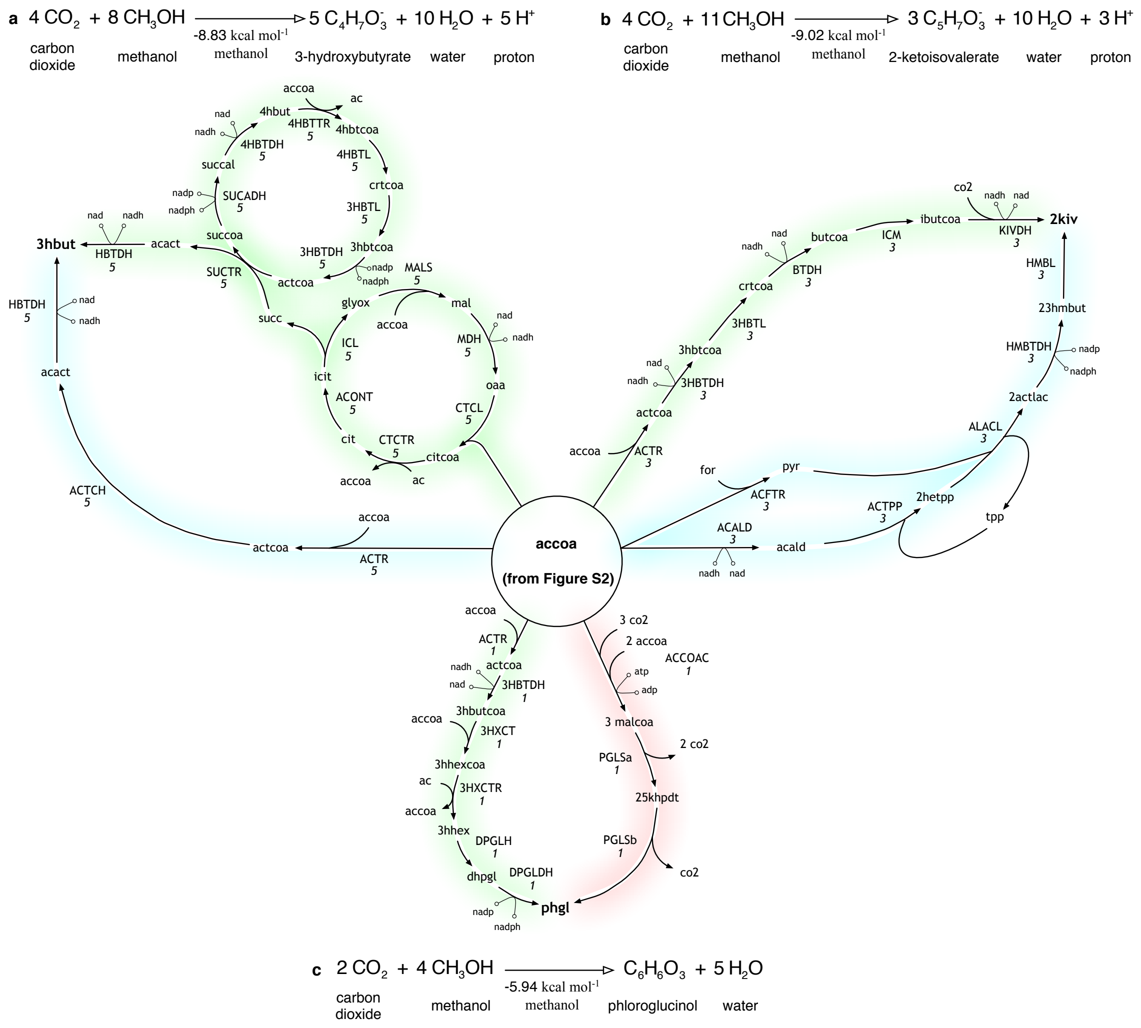

Figure S3: Designs for the co-utilization of methanol and carbon dioxide towards (A) 3-hydroxybutyrate (B) 2-ketoisovalerate and (C) aromatic phloroglucinol. The biosynthetic network for acetyl-CoA (accoa) from methane (CH_4) and carbon dioxide (CO_2) is shown in Figure S2. Metabolic pathways explored in previous studies are highlighted in blue, while proposed ones are highlighted in green. Existing pathway for phloroglucinol production with lower energy efficiency is highlighted in red.

<u>Case Study 3: Designing thermodynamically feasible paths for the conversion of methane to acetate</u>

Similar to Case Study 2, a core set of reactions first converted methane and the co-reactant to the intermediate acetyl-CoA. All designs show that the terminal acceptors exchanged electrons with a cofactor pair instead of directly catalyzing a reaction in the carbon-transfer network. Different cofactors were identified for the electron acceptors depending on the reduction reactions in the

network. For example, Fe^{3+}/Fe^{2+} and NO_2^-/NH_4^+ use nadh as the electron donor, catalyzed by NAD:Fe oxidoreductase (FEDH, from denitrifying bacteria such as *P. denitrificans* [22]) and ammonia oxidoreductase (NH3DH, from *E. coli* [23]) respectively. In contrast, HSO_3^-/H_2S exchanges electrons with napdh using sulfite reductase (HSDH, e.g., in *E. coli* [24]), while reduced ferredoxin does the same for NO_3^-/NO_2^- using a nitrite oxidoreductase (NIFDH, e.g., from *N. gracilis* [25]). In some cases photosynthetic enzymes found in plant chlorophylls were suggested such as ferredoxin-dependent thiosulfate reductase (from *A. thaliana* [26]) for $HSO_3^-/S_2O_3^{2-}$ (see Figure S5c). Upon excluding them an alternate mechanism was identified by minFlux where HSO_3^- is converted to $S_2O_3^{2-}$ through the oxidation of mercaptopyrvate (to pyruvate) by sulfurtransferases found in *E. coli* [27] (see Figure S5c).

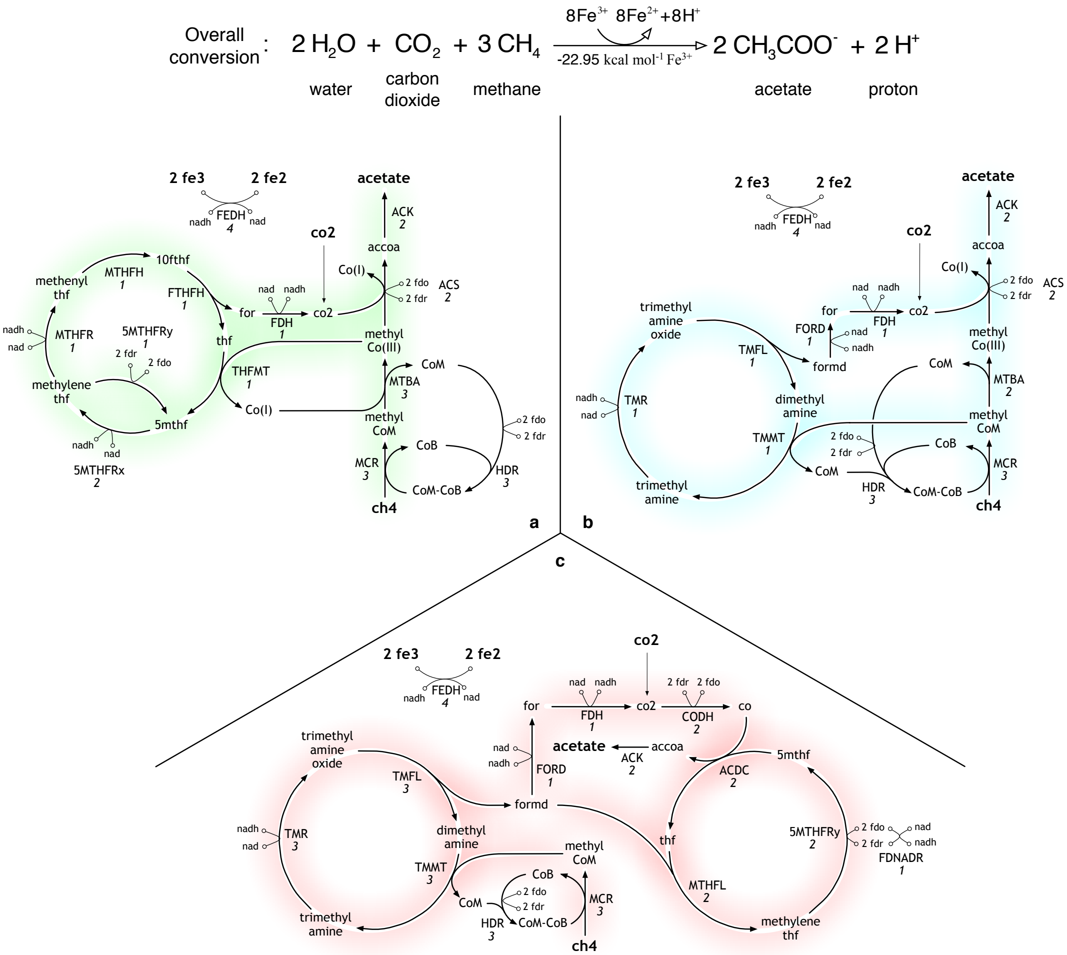

Figure S4: Network designs for fixing methane and carbon dioxide towards acetate using the Fe^{3+}/Fe^{2+} electron acceptor pair.

Figure S4 shows three alternate routes for the conversion of three molecules of methane and one molecule of CO_2 to two acetate molecules with Fe^{3+}/Fe^{2+} as the terminal electron acceptor. The smallest network (see Figure S4a) describes a functional reversal of the methanogenesis pathway where one molecule of methane and CO_2 form acetyl-CoA which is subsequently hydrolyzed to acetate. The key enzymes in this pathway are CoM reductase (MCR), Corrinoid methyltransferase (MTBA), heterodisulfide reductase (HDR) and the acetyl-CoA synthase (ACS) all found in most methanogenic archaea and some methanotrophs [28]. However, in order to maintain the 1:1 ratio of methane and CO_2 in the ACS reaction, one molecule of methane is oxidized to CO_2 in a complex three-step cyclic route involving a functional reversal of the eastern branch of the Wood-Ljungdahl pathway (see Figure S4a). Alternate routes for oxidizing methane were also found that directly oxidize methyl-CoM to CO_2 based on the methylamine cycle (see Figure S4b). The third design (see Figure S4c) bypasses the ACS and MTBA reactions by combining the two cycles describes in Figures S4a and b. Interestingly all the recruited enzymes (except for the terminal electron acceptors) are present in most anaerobic methanogens. This case study demonstrates how cofactor balances can become the dominant factor in synthetic pathway design. For example, the net reduction of 5-methyl thf to 5,10-methylene thf is accomplished by two reactions running in opposite directions involving nad and ferredoxin cofactors (Figure S4a) to maintain overall balance of cofactors.

Figure S5 illustrates a few of the existing (in blue) and newly identified strategies (in green) for the production of four platform chemicals (i.e., 2,3-butanediol, 3-hydroxypropionate, 1-butanol and 1,3-propanediol [29,30]) each utilizing a different electron acceptor. For example, 2,3-butanediol (23but) is produced from acetyl-CoA by first converting it to pyruvate (using pyruvate-ferredoxin oxidoreductase (PFOR) [6]) with subsequent dimerization to 2-acetolactate (alac) (see Figure S5a). Decarboxylation of alac followed by reduction of acetoin (actn) produces 2,3-butanediol. While existing pathways (from pyruvate [31]) lose two carbon atoms for each molecule of 23but, the identified pathway (Figure S5a in blue) preserves all carbon as lost CO_2 is fixed back in the PFOR step. It is interesting to note that the identified pathway uses the same enzymes (except the MTA reaction) recruited for converting CO to 23bdo in a recent study performed on three separate acetogenic *Clostridial* species [32] and functionally expressed in *E. coli* [33]. An alternate pathway was also identified (see Figure S5a in green) where acetyl-CoA is ultimately converted to acetoin through the reversal of acetoin dehydrogenase (ACNDH) found in acetoin consuming bacteria such as *E. aerogenes* [34].

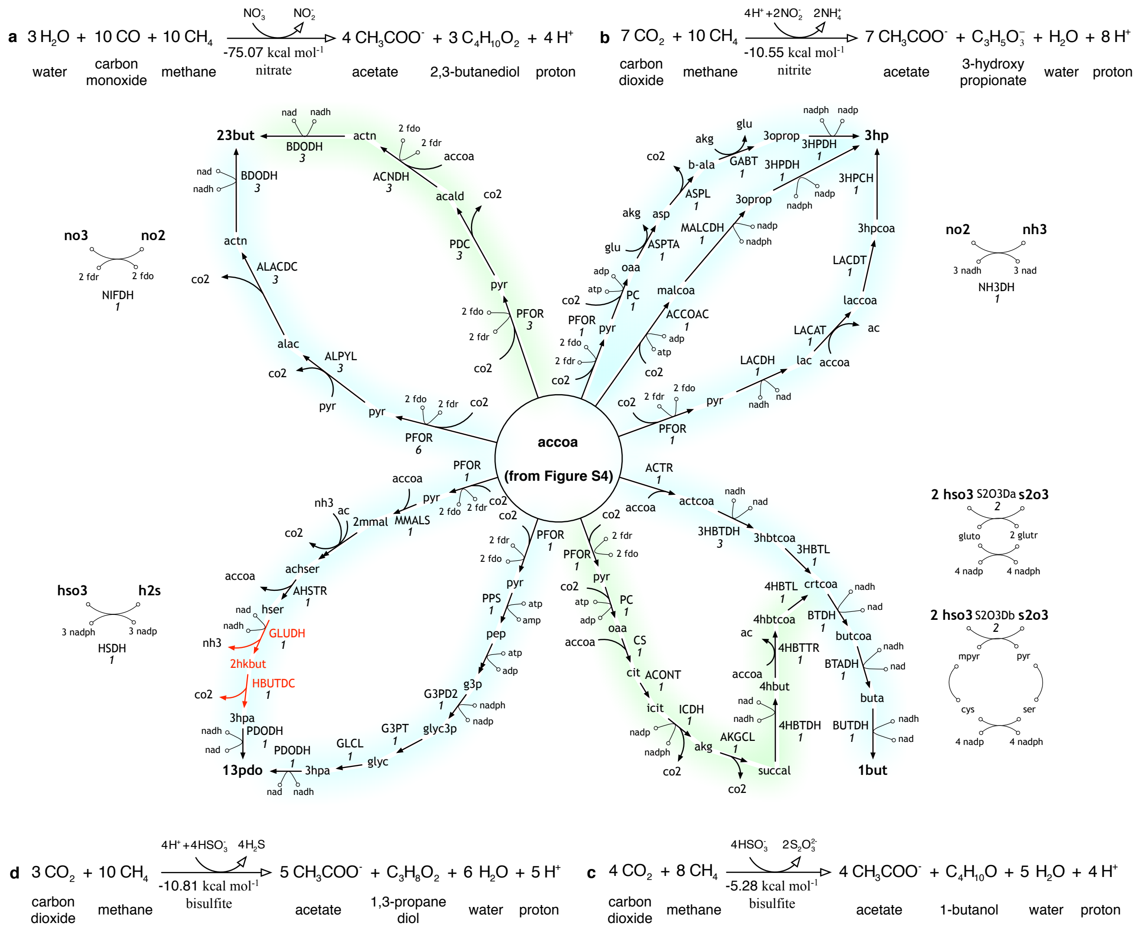

Figure S5: Network designs for conversion of methane and C_1 co-reactant towards four C_{2+} target chemicals. Metabolic network of synthesis of acetyl-CoA (accoa) from methane (CH_4) and co-metabolite (CO_2 or CO) is shown in Figure S4. Metabolic pathways proposed in previous studies are highlighted in blue while new designs are drawn in green.

Three separate pathways were identified for 3-hydroxypropionate (3hp) production all of which have been explored previously [35-37] (see Figure S5b). Similarly, the shortest route for 1-butanol production (Figure S5c) recapitulates existing strategies involving condensation of two molecules of acetyl-CoA by the accoa acetyltransferase (ACTR) followed by a functional reversal of the β-oxidation pathway [38-40]. A so far unexplored pathway is also suggested that combines the oxidative branch of TCA cycle with succinate-semialdehyde metabolism to reach 1-butanol (see Figure S5c in green). For 1,3-propanediol (13pdo) (see Figure S5d), the only identified pathway converts acetyl-CoA to pyruvate which is reduced back to glyceraldehyde-3-phosphate (g3p) through gluconeogenesis. Glycerol, from g3p, is subsequently reduced to 13pdo in three steps by the native in some *Clostridia* [41] or engineered enzymes found in *E. coli* and *K. pneumonie* (i.e.,

the DuPont pathway [42]). Our procedure could not identify an alternate pathway in *E. coli* [43] where homoserine (hser) flux is routed towards 3-hydroxypropanal (3hpa) by engineering the native glutamate dehydrogenase to be promiscuous towards homoserine. The reason is because the intermediate metabolite 4-hydroxy-2-oxobutyrate (4hkbut) in the pathway as well as the reactions associated with the molecule (see Figure S5d in red) were absent in our database. Upon inclusion of the metabolite and the reactions, minFlux could identify this pathway as an alternate more energy efficient route for 13pdo production.

Comparison of optStoic+minRxn/minFlux derived designs with other pathway design tools

We compared the optimal pathways identified by our approach with two existing pathway searching tools (Chou *et al* [44] and Bar-Even *et al* [45]) for all three case studies. The results showed that the graph-based algorithm for Chou *et al* [44], relying on a depth-first search algorithm with no metabolite balances, identified only shortest linear paths connecting the primary substrate to the primary product for all the case studies, while failing to identify any of the carbon-conserving cyclic networks (Figure S6, in blue). The Bar-Even *et al* [45] approach successfully recapitulated the Non Oxidative Glycolysis (NOG) cycle (Case Study 1), however, cofactor imbalances were introduced for the last two studies (Figure S6, in red). The comparison demonstrates that by first fixing an overall stoichiometry (i.e., optStoic), identified reaction designs are focused on only the ones that maximally meet all performance objectives.

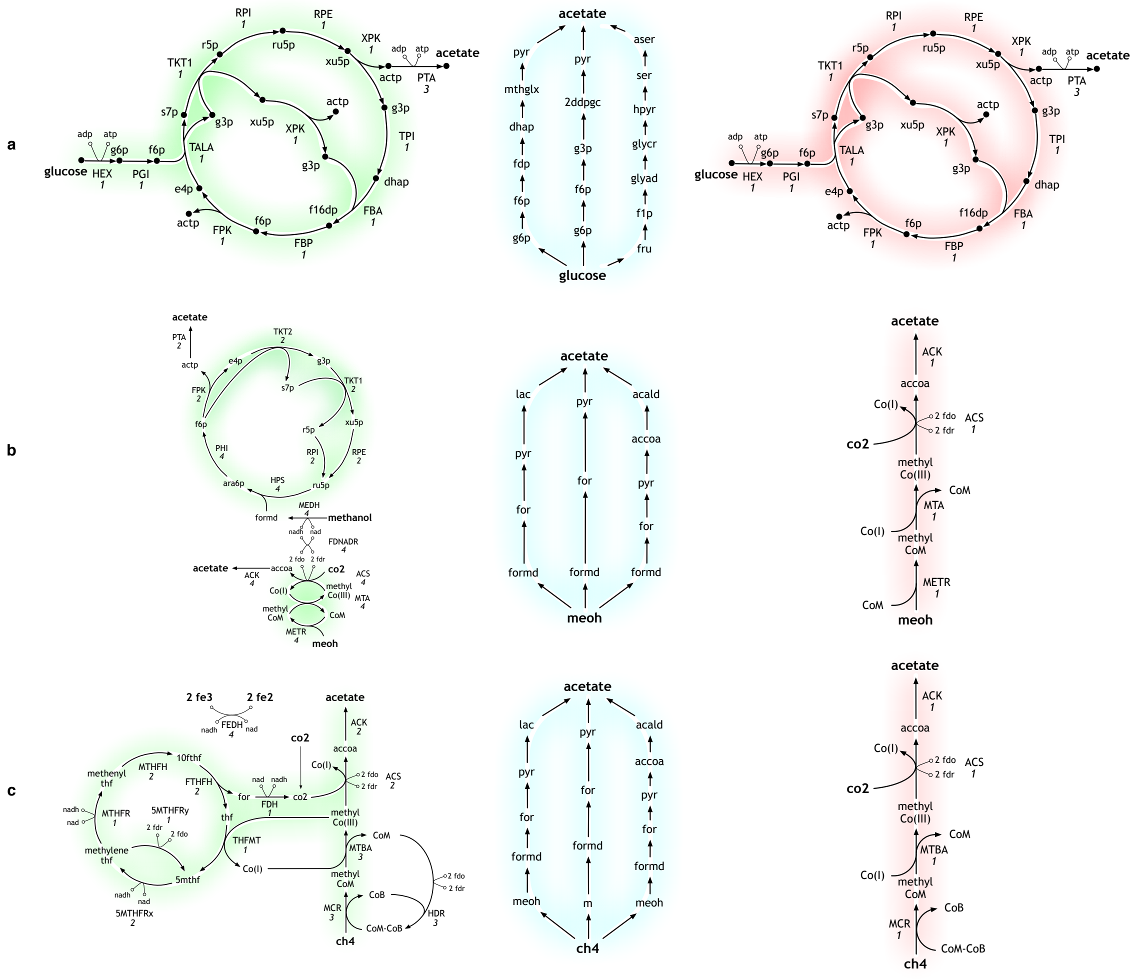

Figure S6. Comparison of the minimal network designs identified by our approach (in green), Chou *et al* [44] (in blue) and Bar-Even *et al* [45] (in red) for the three case studies: (a) conversion of glucose to acetate, (b) co-utilization of methanol and CO_2 to acetate and (c) co-utilization of methane and CO_2 to acetate using electron acceptor pairs.

Reference

1 Bogorad, I. W., Lin, T. S. & Liao, J. C. Synthetic non-oxidative glycolysis enables complete carbon conservation. *Nature* **502**, 693-697, doi:10.1038/nature12575 (2013).

2 Wiegant, W. M. & Debont, J. A. M. A New Route for Ethylene-Glycol Metabolism in Mycobacterium-E44. *J. Gen. Microbiol.* **120**, 325-331 (1980).

3 Gawand, P., Hyland, P., KEknis, A., Martin, V. J. J. & Mahadevan, R. Novel approach to engineer strains for simultaneous sugar utilization. *Metab. Eng.* **20**, 63-72 (2013).

4 Tracy, B. P., Jones, S. W., Fast, A. G., Indurthi, D. C. & Papoutsakis, E. T. Clostridia: the importance of their exceptional substrate and metabolite diversity for biofuel and biorefinery applications. *Curr. Opin. Biotechnol.* **23**, 364-381, doi:10.1016/j.copbio.2011.10.008 (2012).

5 Huang, H., Wang, S., Moll, J. & Thauer, R. K. Electron bifurcation involved in the energy metabolism of the acetogenic bacterium Moorella thermoacetica growing on glucose or H2 plus CO2. *J. Bacteriol.* **194**, 3689-3699, doi:10.1128/JB.00385-12 (2012).

6 Ragsdale, S. W. Pyruvate ferredoxin oxidoreductase and its radical intermediate. *Chem. Rev.* **103**, 2333-2346, doi:10.1021/cr020423e (2003).

7 Kolb, S. & Stacheter, A. Prerequisites for amplicon pyrosequencing of microbial methanol utilizers in the environment. *Frontiers in microbiology* **4**, 268, doi:10.3389/fmicb.2013.00268 (2013).

8 Pritchett, M. A. & Metcalf, W. W. Genetic, physiological and biochemical characterization of multiple methanol methyltransferase isozymes in Methanosarcina acetivorans C2A. *Mol. Microbiol.* **56**, 1183-1194, doi:10.1111/j.1365-2958.2005.04616.x (2005).

9 Doukov, T. I., Iverson, T. M., Seravalli, J., Ragsdale, S. W. & Drennan, C. L. A Ni-Fe-Cu center in a bifunctional carbon monoxide dehydrogenase/acetyl-CoA synthase. *Science* **298**, 567-572, doi:10.1126/science.1075843 (2002).

10 Bogorad, I. W. *et al.* Building carbon-carbon bonds using a biocatalytic methanol condensation cycle. *Proc. Natl. Acad. Sci. U S A* **111**, 15928-15933, doi:10.1073/pnas.1413470111 (2014).

11 Kato, N., Yurimoto, H. & Thauer, R. K. The physiological role of the ribulose monophosphate pathway in bacteria and archaea. *Biosci. Biotechnol. Biochem.* **70**, 10-21, doi:10.1271/bbb.70.10 (2006).

12 Bender, R. A. Regulation of the histidine utilization (hut) system in bacteria. *Microbiol. Mol. Biol. Rev.* **76**, 565-584, doi:10.1128/MMBR.00014-12 (2012).

13 Alissandratos, A., Kim, H. K. & Easton, C. J. Formate production through biocatalysis. *Bioengineered* **4**, 348-350, doi:10.4161/bioe.25360 (2013).

14 Krainer, F. W. *et al.* Recombinant protein expression in Pichia pastoris strains with an engineered methanol utilization pathway. *Microb. Cell Fact.* **11**, 22, doi:10.1186/1475-2859-11-22 (2012).

15 Mainguet, S. E., Gronenberg, L. S., Wong, S. S. & Liao, J. C. A reverse glyoxylate shunt to build a non-native route from C4 to C2 in Escherichia coli. *Metab. Eng.* **19**, 116-127, doi:10.1016/j.ymben.2013.06.004 (2013).

16 Hagishita, T., Yoshida, T., Izumi, Y. & Mitsunaga, T. Cloning and expression of the gene for serine-glyoxylate aminotransferase from an obligate methylotroph Hyphomicrobium methylovorum GM2. *Eur. J. Biochem.* **241**, 1-5 (1996).
17 Heinrich, D., Raberg, M. & Steinbuchel, A. Synthesis of poly(3-hydroxybutyrate-co-3-hydroxyvalerate) from unrelated carbon sources in engineered Rhodospirillum rubrum. *FEMS Microbiol. Lett.*, doi:10.1093/femsle/fnv038 (2015).
18 Zhang, J., Gao, X., Hong, P. H., Li, Z. J. & Tan, T. W. Enhanced production of poly-3-hydroxybutyrate by Escherichia coli over-expressing multiple copies of NAD kinase integrated in the host genome. *Biotechnol. Lett.*, doi:10.1007/s10529-015-1797-1 (2015).
19 Buchholz, J. *et al.* Platform engineering of Corynebacterium glutamicum with reduced pyruvate dehydrogenase complex activity for improved production of L-lysine, L-valine, and 2-ketoisovalerate. *Appl. Environ. Microbiol.* **79**, 5566-5575, doi:10.1128/AEM.01741-13 (2013).
20 Cracan, V. & Banerjee, R. Novel coenzyme B12-dependent interconversion of isovaleryl-CoA and pivalyl-CoA. *J. Biol. Chem.* **287**, 3723-3732, doi:10.1074/jbc.M111.320051 (2012).
21 Yang, F. & Cao, Y. Biosynthesis of phloroglucinol compounds in microorganisms--review. *Appl. Microbiol. Biotechnol.* **93**, 487-495, doi:10.1007/s00253-011-3712-6 (2012).
22 Mazoch, J., Tesarik, R., Sedlacek, V., Kucera, I. & Turanek, J. Isolation and biochemical characterization of two soluble iron(III) reductases from Paracoccus denitrificans. *Eur. J. Biochem.* **271**, 553-562 (2004).
23 Jackson, R. H., Cornish-Bowden, A. & Cole, J. A. Prosthetic groups of the NADH-dependent nitrite reductase from Escherichia coli K12. *Biochem. J.* **193**, 861-867 (1981).
24 Murphy, M. J., Siegel, L. M. & Kamin, H. Reduced nicotinamide adenine dinucleotide phosphate-sulfite reductase of enterobacteria. VI. The reaction of carbon monoxide with the Escherichia coli holoenzyme, the hemoprotein, and free siroheme. *J. Biol. Chem.* **249**, 1610-1614 (1974).
25 Lucker, S., Nowka, B., Rattei, T., Spieck, E. & Daims, H. The Genome of Nitrospina gracilis Illuminates the Metabolism and Evolution of the Major Marine Nitrite Oxidizer. *Frontiers in microbiology* **4**, 27, doi:10.3389/fmicb.2013.00027 (2013).
26 Setya, A., Murillo, M. & Leustek, T. Sulfate reduction in higher plants: molecular evidence for a novel 5'-adenylylsulfate reductase. *Proc. Natl. Acad. Sci. U S A* **93**, 13383-13388 (1996).
27 Vachek, H. & Wood, J. L. Purification and properties of mercaptopyruvate sulfur transferase of Escherichia coli. *Biochim. Biophys. Acta* **258**, 133-146 (1972).
28 Mueller, T. J. *et al.* Methane oxidation by anaerobic archaea for conversion to liquid fuels. *J. Ind. Microbiol. Biotechnol.* **42**, 391-401, doi:10.1007/s10295-014-1548-7 (2015).
29 de Jong, E., Higson, A., Walsh, P. & Wellisch, M. Bio-based chemicals value added products from biorefineries. (IEA Bioenergy, Task42 Biorefinery, 2012).

30 Jang, Y. S. *et al.* Bio-based production of C2-C6 platform chemicals. *Biotechnol. Bioeng.* **109**, 2437-2459, doi:10.1002/bit.24599 (2012).
31 Xiao, Z. & Lu, J. R. Strategies for enhancing fermentative production of acetoin: a review. *Biotechnol. Adv.* **32**, 492-503, doi:10.1016/j.biotechadv.2014.01.002 (2014).
32 Kopke, M. *et al.* 2,3-butanediol production by acetogenic bacteria, an alternative route to chemical synthesis, using industrial waste gas. *Appl. Environ. Microbiol.* **77**, 5467-5475, doi:10.1128/AEM.00355-11 (2011).
33 Kopke, M. *et al.* Reconstruction of an acetogenic 2,3-butanediol pathway involving a novel NADPH-dependent primary-secondary alcohol dehydrogenase. *Appl. Environ. Microbiol.* **80**, 3394-3403, doi:10.1128/AEM.00301-14 (2014).
34 Carballo, J., Martin, R., Bernardo, A. & Gonzalez, J. Purification, characterization and some properties of diacetyl(acetoin) reductase from Enterobacter aerogenes. *Eur. J. Biochem.* **198**, 327-332 (1991).
35 Borodina, I. *et al.* Establishing a synthetic pathway for high-level production of 3-hydroxypropionic acid in Saccharomyces cerevisiae via beta-alanine. *Metab. Eng.* **27**, 57-64, doi:10.1016/j.ymben.2014.10.003 (2015).
36 Honjo, H., Tsuruno, K., Tatsuke, T., Sato, M. & Hanai, T. Dual synthetic pathway for 3-hydroxypropionic acid production in engineered Escherichia coli. *J. Biosci. Bioeng.*, doi:10.1016/j.jbiosc.2014.12.023 (2015).
37 Rathnasingh, C. *et al.* Production of 3-hydroxypropionic acid via malonyl-CoA pathway using recombinant Escherichia coli strains. *J. Biotechnol.* **157**, 633-640, doi:10.1016/j.jbiotec.2011.06.008 (2012).
38 Atsumi, S. *et al.* Metabolic engineering of Escherichia coli for 1-butanol production. *Metab. Eng.* **10**, 305-311, doi:10.1016/j.ymben.2007.08.003 (2008).
39 Dellomonaco, C., Clomburg, J. M., Miller, E. N. & Gonzalez, R. Engineered reversal of the beta-oxidation cycle for the synthesis of fuels and chemicals. *Nature* **476**, 355-359, doi:10.1038/nature10333 (2011).
40 Lim, J. H., Seo, S. W., Kim, S. Y. & Jung, G. Y. Model-driven rebalancing of the intracellular redox state for optimization of a heterologous n-butanol pathway in Escherichia coli. *Metab. Eng.* **20**, 56-62, doi:10.1016/j.ymben.2013.09.003 (2013).
41 Leja, K. *et al.* Hypothetical glycerol pathways of newly isolated strains capable of 1,3-propanediol production. *Acta Biochim. Pol.* **61**, 759-763 (2014).
42 Emptage, M., Haynie, S. L., Laffend, L. A., Pucci, J. P. & Whited, G. Genetically engineered Escherichia coli containing nonspecific dehydratase yghD and dha regulon for the biological production of 1, 3-propanediol with high titer. US Patent patent (1997).
43 Celinska, E. Fully glycerol-independent microbial production of 1, 3-propanediol via non-natural pathway: Paving the way to success with synthetic tiles. *Biotechnol. J.*, doi:10.1002/biot.201400360 (2014).
44 Chou, C. H., Chang, W. C., Chiu, C. M., Huang, C. C. & Huang, H. D. FMM: a web server for metabolic pathway reconstruction and comparative analysis. *Nucleic Acids Res.* **37**, W129-134, doi:10.1093/nar/gkp264 (2009).

45 Bar-Even, A., Noor, E., Lewis, N. E. & Milo, R. Design and analysis of synthetic carbon fixation pathways. *Proc. Natl. Acad. Sci. U S A* **107**, 8889-8894, doi:10.1073/pnas.0907176107 (2010).
